# Tumor extracellular matrix modulating strategies for enhanced antitumor therapy of nanomedicines

**DOI:** 10.1016/j.mtbio.2022.100364

**Published:** 2022-07-15

**Authors:** Meng Li, Yijing Zhang, Qin Zhang, Jingchao Li

**Affiliations:** a State Key Laboratory for Modification of Chemical Fibers and Polymer Materials, College of Biological Science and Medical Engineering, Donghua University, Shanghai, 201620, China; b Institute of Translational Medicine, Shanghai University, Shanghai, 200444, PR China

**Keywords:** Extracellular matrix, Cancer therapy, Nanomedicines, Tumor accumulation, Immunotherapy

## Abstract

Nanomedicines have shown a promising strategy for cancer therapy because of their higher safety and efficiency relative to small-molecule drugs, while the dense extracellular matrix (ECM) in tumors often acts as a physical barrier to hamper the accumulation and diffusion of nanoparticles, thus compromising the anticancer efficacy. To address this issue, two major strategies including degrading ECM components and inhibiting ECM formation have been adopted to enhance the therapeutic efficacies of nanomedicines. In this review, we summarize the recent progresses of tumor ECM modulating strategies for enhanced antitumor therapy of nanomedicines. Through degrading ECM components or inhibiting ECM formation, the accumulation and diffusion of nanoparticles in tumors can be facilitated, leading to enhanced efficacies of chemotherapy and phototherapy. Moreover, the ECM degradation can improve the infiltration of immune cells into tumor tissues, thus achieving strong immune response to reject tumors. The adoptions of these two ECM modulating strategies to improve the efficacies of chemotherapy, phototherapy, and immunotherapy are discussed in detail. A conclusion, current challenges and outlook are then given.

## Highlights

• Extracellular matrix modulating strategies have been adopted to enhance the therapeutic efficacies of nanomedicines.
• Degrading extracellular matrix components or inhibiting extracellular matrix formation can improve the accumulation and diffusion of nanoparticles in tumors and the infiltration of immune cells into tumor tissues.
• The adoptions of two extracellular matrix modulating strategies to improve the efficacies of chemotherapy, phototherapy, and immunotherapy are summarized.

## Graphical abstract

**Figure:**
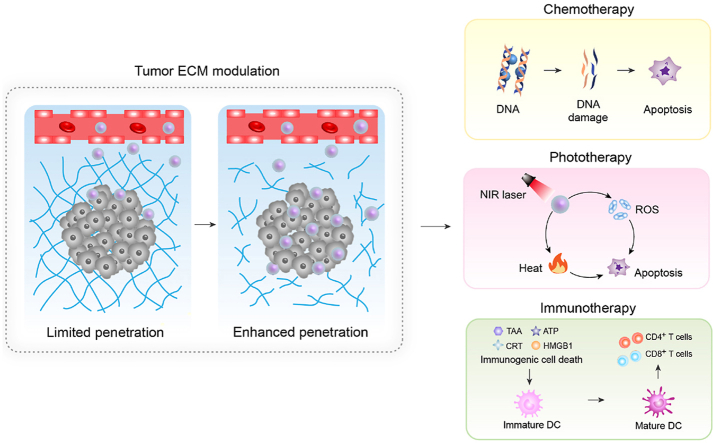

## 1 Introduction

Effective treatments of cancer remain a major challenge in clinical oncology [1]. Conventional methods including surgery, chemotherapy, and radiotherapy often have some limitations and fail to achieve ideal therapeutic goals [[2], [3], [4]]. Surgery has the problem of incomplete excision that will lead to tumor recurrence [5]. Chemotherapy and radiotherapy encounter the predicaments of low therapeutic efficacy, severe side effect and therapy resistance [[6], [7], [8]]. Recently, nanomedicines have been developed to improve the efficacy and safety of cancer therapy [[9], [10], [11]]. The utilizations of nanoplatforms as drug nanocarriers can improve the pharmacokinetics of small-molecular anticancer drugs, and mediate their delivery into tumor sites, which not only allows enhanced anticancer efficacies, but also reduces the side effects [[12], [13], [14]]. Through producing toxic signals, such as heat and reactive oxygen species (ROS), nanomedicines themselves can serve as therapeutic agents for cancer treatment [[15], [16], [17]]. In addition, nanoplatforms can enable the integrations of different therapeutic components into single systems to achieve combinational therapy [[18], [19], [20]].

Although nanomedicines have provided an alternative approach for cancer treatment, the dense extracellular matrix (ECM) in tumors can establish a physical barrier to hinder the accumulation and diffusion of nanotherapeutics at the tumor sites, thus greatly compromising the therapeutic efficacies [[21], [22], [23], [24], [25], [26]]. Some solid tumors, such as breast cancer, pancreatic cancers, and bladder cancers often consist of abundant ECM [27]. The ECM components in tumors such as collagen and hyaluronic acid (HA) mainly secreted by stromal cells are highly dysregulated and cross-linked to form a complex network [[28], [29], [30]]. Recent studies have shown that the degradation of tumor ECM and inhibition of tumor ECM formation can promote the accumulation and penetration of nanotherapeutics at tumor sites, and thus the therapeutic efficacies of nanomedicines can be improved [21]. In addition, the dense ECM hinders the infiltration of immune cells into tumor tissues to limit the antitumor effect of immunotherapy [[31], [32], [33]]. To solve this problem, the compactness of ECM can be reduced via degrading the main components of ECM, and thus the permeability of tumor tissues can be improved to facilitate the migrations and infiltrations of immune cells into tumors [34]. Therefore, modulation of ECM in tumors not only contributes to the improved enrichments and diffusions of nanomedicines, but also facilitates the infiltrations of immune cells in tumor tissues, thereby enhancing the antitumor effect.

**Fig. 1:**
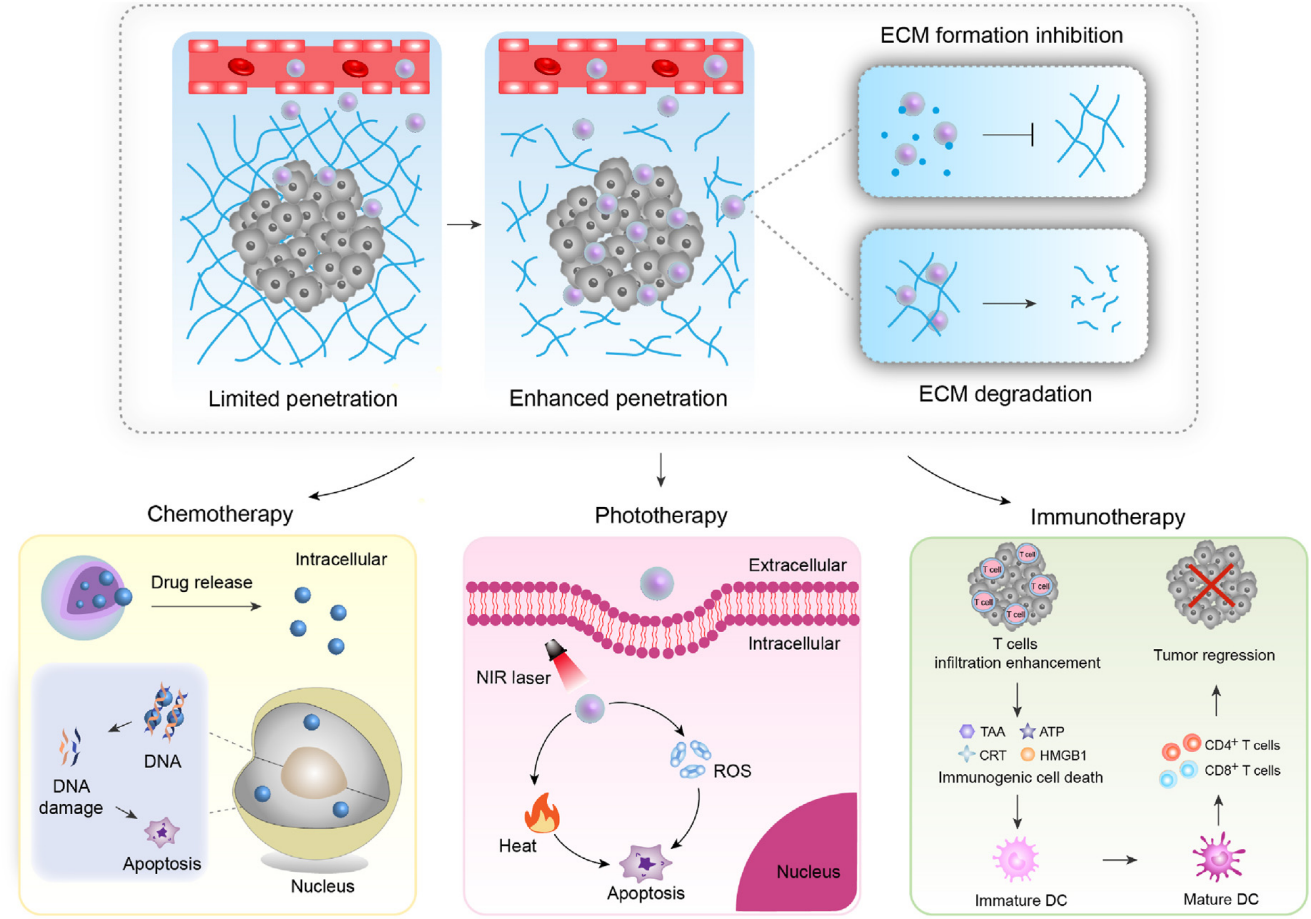
Summary of ECM modulating strategies for enhanced chemotherapy, phototherapy and immunotherapy using nanomedicines.

In this review, the recent progresses of tumor ECM modulating strategies for enhanced cancer therapy of nanomedicines are summarized (Fig. 1). In the following sections, degradation of tumor ECM and inhibition of tumor ECM formation to improve the efficacies of nanomedicine-mediated chemotherapy, phototherapy, and immunotherapy of tumors are introduced, respectively. Then, a brief conclusion of this review and future perspectives of ECM modulating strategies for cancer therapy are discussed.

## 2 ECM modulation for enhanced chemotherapy

### 2.1 Tumor ECM degrading strategy

Due to the abundant collagen in tumor ECM, some enzymes that can degrade collagen have been used to modulate tumor ECM for enhanced chemotherapy. As an example, Tang's group developed a bromelain-immobilized and lactobionic acid (LA)-modified chitosan nanoparticle to improve the drug penetration in tumor tissues for enhanced chemotherapy [35]. Such dual-functional nanoparticles were used as nanocarriers for doxorubicin (DOX), in which, bromelain possessed a proteolytic activity to degrade tumor ECM and thus enhanced the permeation and diffusion of DOX-loaded nanoparticles in tumors, and LA further increased the nanoparticle accumulation via active tumor-targeting mechanism. As such, the dual-functional chitosan nanoparticles could perform superior targeting and permeation ability within tumor tissues, leading to higher drug concentration in tumor area and superior antitumor effect relative to control nanoparticles without modification of bromelain in H22 tumor-bearing mice. Recently, the same group constructed a pH-sensitive bromelain nanoparticle to facilitate penetration of DOX in solid tumors for enhanced chemotherapy [36]. The pH-sensitive nanocarriers were prepared via crosslinking of bromelain using an ortho ester-based crosslink agent, followed by encapsulation of DOX into nanoparticles. The formed nanoparticles exhibited pH-responsive DOX release due to their degradation in mildly acidic condition, and the released bromelain enabled destruction of tumor ECM to further promote nanoparticle penetration in tumor sites. In addition, bromelain inhibited the growth of tumor cells at high concentration, synergizing with DOX to cause antitumor effects. Therefore, such bromelain-based pH-sensitive nanoparticles delivered more drugs into tumors and led to a high tumor growth inhibition of 62.5% for H22 tumors.

Collagenase is a water-soluble matrix metalloprotease that can specifically degrade collagen, and thus has be used for tumor ECM modulation [37]. For example, Cao's group reported the construction of collagenase IV and clusterin-modified polycaprolactone-polyethylene glycol (PEG) nanoparticles with loading of DOX for enhanced cancer chemotherapy [38]. Such nanoparticles not only exhibited reduced phagocytosis by the reticuloendothelial system, but also showed increased penetration ability through 2D and 3D ECM models via collagenase IV-mediated tumor ECM degradation. Thus, the nanoparticles obviously reduced the distribution of DOX in normal organs, but significantly increased DOX accumulation in tumor tissues, leading to remarkable antitumor effect to inhibit the growth of MCF tumors in mouse models. Tang's group reported a hybrid pH-sensitive alginate-based and ortho ester-contained nanogel with surface functionalization of collagenase as DOX carriers for enhanced cancer chemotherapy [39]. The pH-triggered swelling and degradation of ortho ester components in mildly acidic environments led to sustained release of DOX. Via collagenase-mediated degradation of tumor ECM, the collagenase-functionalized nanogels showed higher penetration ability and delivered more DOX into tumor area relative to control counterparts, leading to a better antitumor efficacy in inhibiting the growth of H22 tumors.

**Fig. 2:**
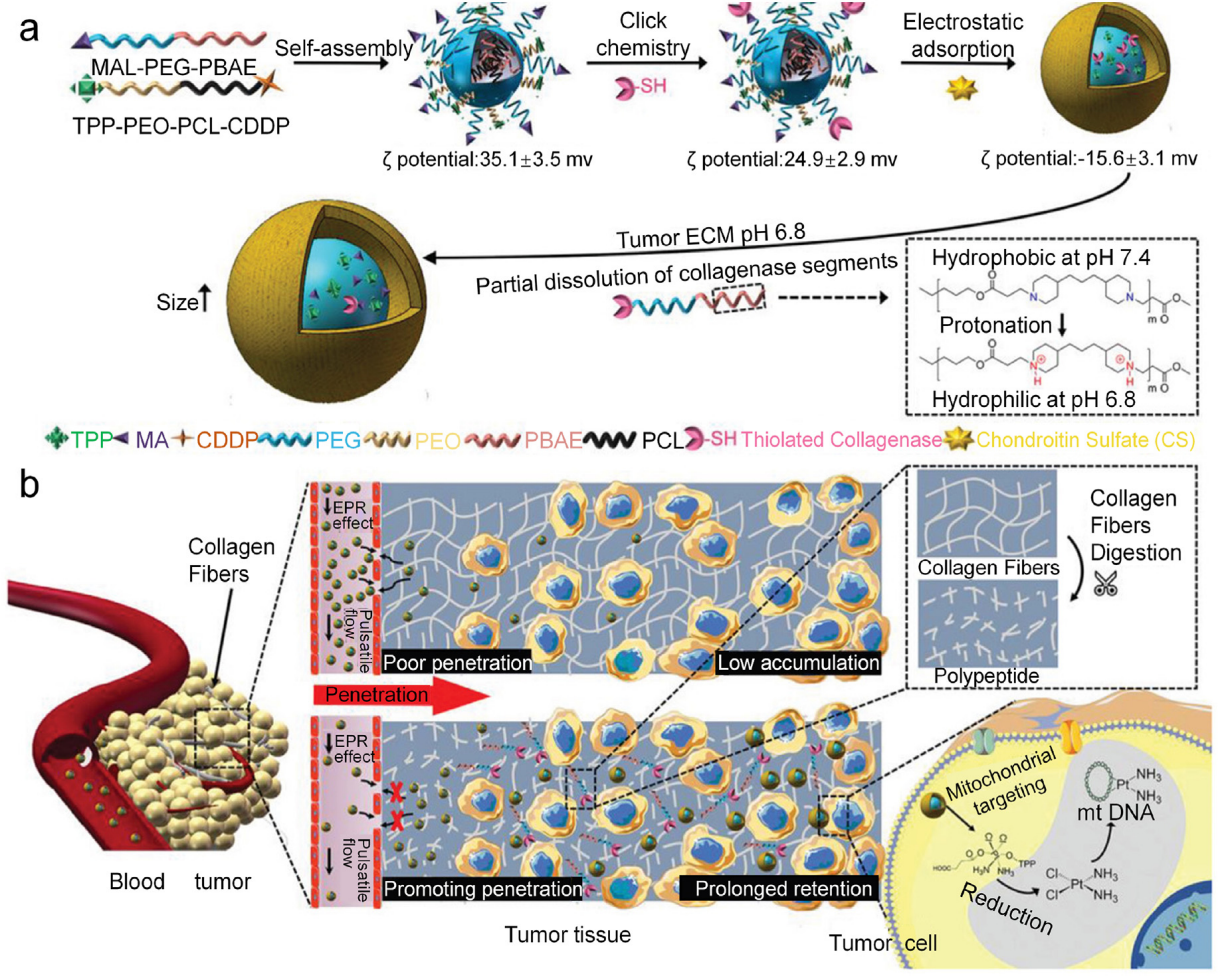
(a) Schematic illustration of the fabrication of collagenase-based nanoscavengers and their size increase and dissociation of the collagenase containing components in response to the acidic pH. (b) Schematic illustration of the increased penetration and retention of nanoparticles in deep tumor tissues via the combined action of collagenase digestion of collagen fibers and particle size increase, and the destruction of mitochondrial DNA via mitochondria-specific targeting and release of cisplatin drugs into mitochondria. Reproduced with permission from Ref. [40]. Copyright 2020, Wiley-VCH.

In another study, Zhou's group reported a size-changeable collagenase-modified nanoscavenger to increase the penetration and retention of nanomedicine in deep tumor tissues for chemotherapy [40]. A self-assembled polymer micelle was modified with collagenase through click chemistry, followed by an electrostatic adsorption of chondroitin sulfate as a shell layer to avoid inactivation of collagenase in blood circulation and mask the surface positive charges of nanoparticles (Fig. 2a). Upon acidic stimulation (pH ​= ​6.8) in the tumor microenvironment, the collagenase-containing components on the nanoscavengers changed from hydrophobic to hydrophilic via the protonation of tertiary amine groups to allow partial dissolution of collagenase segments. The collagenase segments acted as scavengers to digest collagen fibers of tumor tissues, greatly enhancing the penetration of nanocarriers (Fig. 2b). The remaining components in the nanocarriers caused the nanoscavenger size expanding to improve their intratumoral retention. Such an enhanced penetration and retention mechanism resulted in a significantly improved accumulation of anticancer agent in tumors. In addition, triphenylphosphonium (TPP) within nanoscavengers could achieve mitochondria targeting and release of cisplatin drugs in mitochondria to destroy the mitochondrial deoxyribonucleic acid (DNA) for killing of cancer cells. As a consequence, the nanoscavenger-mediated chemotherapy showed an excellent anticancer efficacy and could greatly inhibit the growth of 4T1 tumors.

To maintain the active sites of enzyme, Du's group reported a mild acid-responsive “nanoenzyme capsule” through combining collagenase nanocapsules with DOX-encapsulated heavy-chain ferritin nanocages for cancer chemotherapy [41]. Collagenase nanocapsules protected the activity of enzymes, which were degraded in mildly acidic tumor microenvironment to release collagenase, resulting to the digestion of collagen in tumor ECM. This allowed improved accumulation and penetration of DOX-encapsulated nanoparticles in solid tumors and alleviated hypoxia inside the tumor to enhance the antitumor effects of DOX. As such, the nanoenzyme capsule-mediated chemotherapy could inhibit the growth of 4T1 tumors by 85%.

In view of the abundant content of hyaluronic acid (HA) in tumor ECM, hyaluronidase (HAase) that can specifically degrade HA can also be utilized to modulate tumor ECM to enhance penetration of drug-loaded nanoparticles for enhanced chemotherapy [42]. Shi's group reported a HAase-modified 2D layered double hydroxide (LDH) nanodisk with loading of DOX for enhanced tumor penetration and augmented chemotherapy [43]. LDH nanodisks formed via a co-precipitation were functionalized with HAase by electrostatic attraction, which were then used as carriers to load DOX. Such a formed DOX/LDH-HAase nanodisk showed a pH-responsive DOX release profile with a faster release rate in acidic tumor microenvironment. The surface HAase enabled digestion of HA in tumor ECM to allow obviously improved penetration of DOX/LDH-HAase into tumors. The DOX/LDH-HAase-mediated chemotherapy showed an enhanced efficacy in inhibiting the growth of SW1990 tumors. In another similar study, Sun and coworkers developed a HAase-functionalized drug-loaded micelle to increase solid tumor penetration and antitumor efficacy [44]. Anionic HAase was functionalized onto surface of cationic epirubicin-loaded micelles through electrostatic interaction. The polymer segments within micelles showed a pH-sensitive property to allow pH-responsive drug release in lysosome. The surface HAase degraded HA in tumor ECM to improve the penetration efficacy of epirubicin-loaded micelles, leading to their enhanced accumulation and deep tumor penetration in HepG2 tumors. In view of the combinational action of HAase-enabled deep tumor penetration and pH-responsive drug release, this micelle-based chemotherapy showed an enhanced inhibition for the growth of HepG2 tumors.

To achieve enhanced tumor penetration and precise and rapid drug release at tumor sites, Li's group developed a reduction/oxidation-responsive hierarchical nanoparticle with co-encapsulation of paclitaxel (PTX) and pH-stimulated HAase for precise cancer chemotherapy [45]. A HA-stearic acid amphiphilic conjugate containing disulfide bond was used to fabricate the hierarchical nanoparticles, which were used to entrap PTX and pH-stimulated HAase. In slightly acidic tumor microenvironment, pH-stimulated HAase was partially activated to mediate the degradation of tumor ECM for deep tumor penetration of nanoparticles. Via CD44-mediated endocytosis, the nanoparticles specifically bound to CD44 receptor on the cancer cell membrane to promote their cellular uptake. The release of PTX from nanoparticles was accelerated under intracellular redox microenvironment within endo/lysosomes due to the destruction of nanoparticles by the completely activated pH-stimulated HAase. As such, these nanoparticles afforded the highest tumor inhibition (93.71%) in MDA-MB-231 tumor-bearing mouse models.

**Fig. 3:**
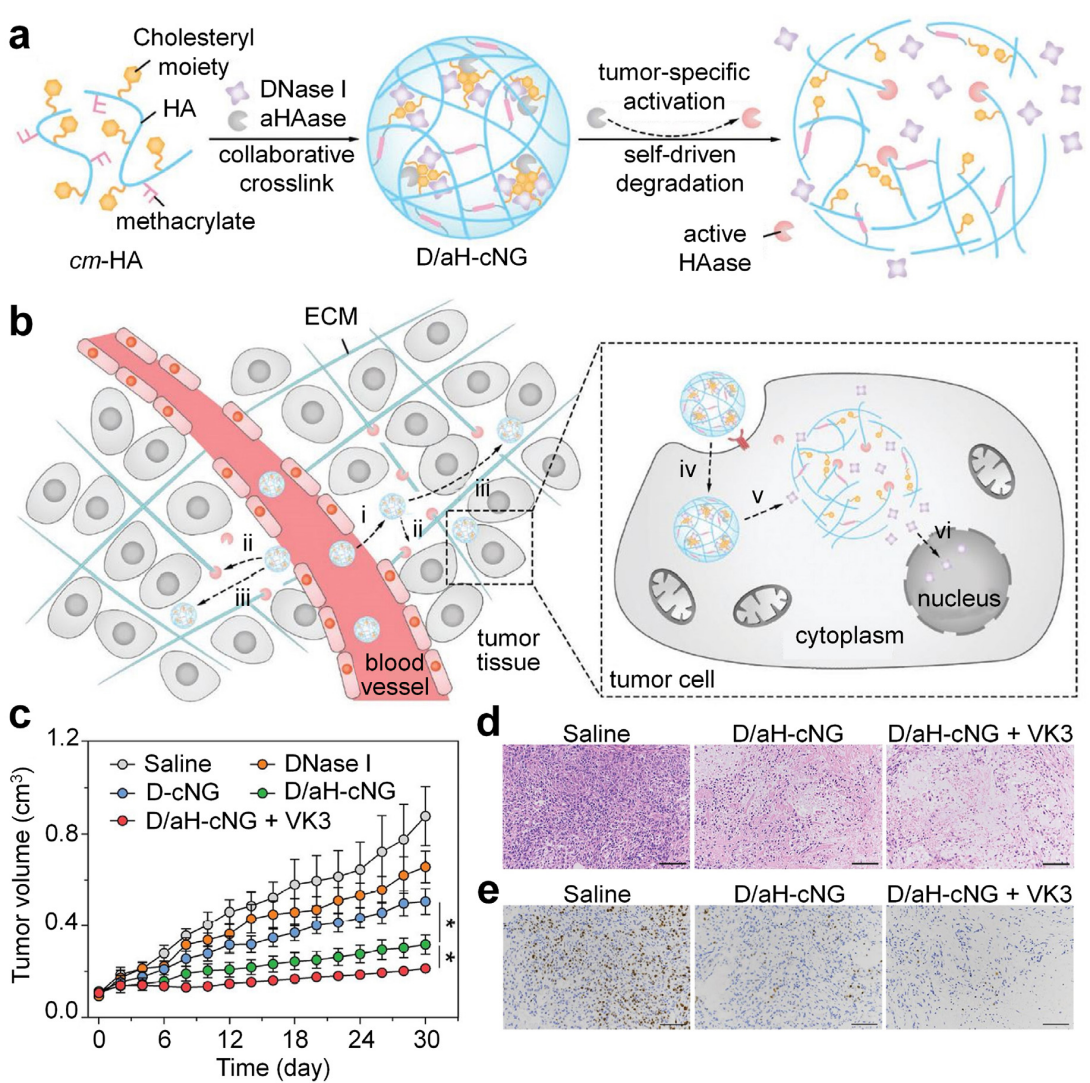
(a) Schematic of self-assembly and tumor-specific self-degradation of the collaboratively crosslinked D/aH-cNG. (b) Schematic of enhanced protein delivery by D/aH-cNG for cancer therapy. (i) D/aH-cNG accumulate into tumor; (ii) slight tumor acidity partially activates aHAase, which leads to the swelling of the D/aH-cNG and the extracellular release of active HAase to degrade the HA-composing ECM for enhanced diffusion in the stroma. (iii) D/aH-cNG penetrate to the deep tumor region. (iv) D/aH-cNG are internalized by the tumor cell; (v) endocytic acidity fully activates aHAase, which causes the complete degradation of the cNG and the rapid intracellular release of DNase I; (vi) the released DNase I digests the DNA to cause the tumor cell death. (c) Tumor size variation of the tumor-bearing nude mice treated with different formulations. (d) Images of the tumors stained with H&E 30 ​d after initial treatment with different formulations. (e) Images of the tumors stained with the Ki-67 antibody 30 ​d after initial treatment with different formulations. Reproduced with permission from Ref. [46]. Copyright 2018, Wiley-VCH.

HAase-mediated tumor ECM degradation has also been used to increase the penetration of toxic proteins for cancer therapy. As an example, Mo's group constructed a self-driven degradable nanogel with acidity-responsive action for efficient delivery of deoxyribonuclease I (DNase I) and enhanced antitumor efficacy [46]. Cholesteryl-6-aminohexylcarbamate methacrylated HA (cm-HA) was used to form collaboratively crosslinked nanogels (cNG) via both physical and chemical crosslinking to load DNase I and a tumor-acidity-activatable HAase (aHAase, the amino residues in HAase were shielded by the citraconic anhydride modification). The DNase I and inactive aHAase loaded cNG (designated as D/aH-cNG) could accumulate into tumor sites, and the extracellular slightly acidic condition could activate aHAase partially and the cNG underwent a self-driven degradation to allow release of aHAase in the stroma (Fig. 3a). The released reactivated aHAase mediated the degradation of tumor ECM to increase the diffusion of cNG into the deep region of tumors. The internalization of D/aH-cNG was improved via CD44 receptor-mediated endocytotic pathway (Fig. 3b). In acidic endocytic vesicles, cNG was completely disintegrated to release DNase I for causing tumor cell death. Therefore, a high inhibitory ratio (62%) of A549 tumors was observed after D/aH-cNG-mediated therapy, and antitumor efficacy was further reinforced in combination with vitamin K3 (VK3) that could activate endogenous DNase I to degrade DNA (Fig. 3c–e).

### 2.2 Tumor ECM formation inhibiting strategy

Cancer-associated fibroblasts (CAFs) are the major component of the stromal cells in tumor sites to secrete mass ECM components [47]. Therefore, suppression of CAF activity can lead to inhibition of tumor ECM production, thus contributing to improved penetration of drugs and nanotherapeutics for enhanced cancer chemotherapy [48]. Chen's group constructed a CAF-targeting biodegradable polymer nanoparticle coated with CREKA peptide and loaded with α-mangostin (α-M) to modulate tumor ECM for cancer therapy [49]. Surface CREKA peptide allowed the targeting of nanoparticles to CAFs via the surface overexpressed fibronectin. The nanoparticles could effectively inactive CAFs, reduce the production of tumor ECM, and facilitate tumor vascular normalization at tumor sites. Thus, the uptake and penetration of gemcitabine and triptolide-loaded micelles modified with CRPPR peptide in orthotopic pancreatic tumors were obviously improved after pretreatment of α-M-loaded nanoparticles. This nanoparticle-mediated tumor ECM modulation enhanced the therapeutic efficacies of both gemcitabine and nanotherapeutics. Huang and coworkers reported a hydralazine (HDZ)-based liposome to promote nanoparticle penetration in advanced desmoplastic tumors for chemotherapy [50]. In murine models of desmoplastic melanoma, the tumor ECM was significantly reduced after three injections of HDZ-liposomes, leading to increased nanoparticle accumulation and penetration inside desmoplastic tumors. As a result, the HDZ-liposome pretreatment remarkably improved the antitumor efficacy of DOX-liposomes in desmoplastic tumors.

Golgi apparatus within hepatic stellate cells (HSCs) play key roles for the production of ECM in liver cancer, and thus the destruction of Golgi apparatus in HSCs would reduce ECM formation and promote the penetration of cargos in tumors [51]. Ma's group constructed a chondroitin-modified lipid nanoparticle with loading of DOX and retinoic acid (RA) to target Golgi apparatus and degrade ECM for chemotherapy of liver cancer [52]. The surface modification of chondroitin enabled the targeting of nanoparticles to both hepatoma cells and HSCs via CD44 receptor-mediated internalization. Thus, the nanoparticles could be efficiently taken up by SMMC-7721 hepatoma cells and HSCs, in which, these nanoparticles released RA to destroy the Golgi apparatus and inhibit ECM production. As such, the nanoparticles remarkably improved the accumulation and penetration of DOX and RA into tumors, showing high antitumor efficacy in mice with primary liver cancer and H22 allograft tumors.

Pancreatic stellate cells (PSCs) are the most critical stroma cells in establishing tumor ECM for pancreatic ductal adenocarcinoma (PDAC) as they secrete excessive ECM proteins [53]. Therefore, inhibition of the activity of PSCs provides an alternative strategy for enhanced chemotherapy. Gao's group recently reported a two-step sequential delivery strategy for enhanced chemotherapy of PDAC [54]. Metformin (MET) that could suppress the activity of PSCs was first administrated into tumor-bearing mice to disrupt the formation of dense tumor ECM via inhibiting the generation of α-smooth muscle actin and collagen, leading to enhanced accumulation and delivery of gemcitabine and pH (low) insertion peptide (pHLIP) co-modified magnetic nanoparticles into tumor sites. The pHLIP increased the binding affinity of nanoparticles to pancreatic cancer cells (PANC-1), in which, gemcitabine was released in response to cathepsin B for cancer cell killing. The combination of MET and nanoparticles could greatly inhibit the growths of both subcutaneous and orthotopic pancreatic tumors.

**Fig. 4:**
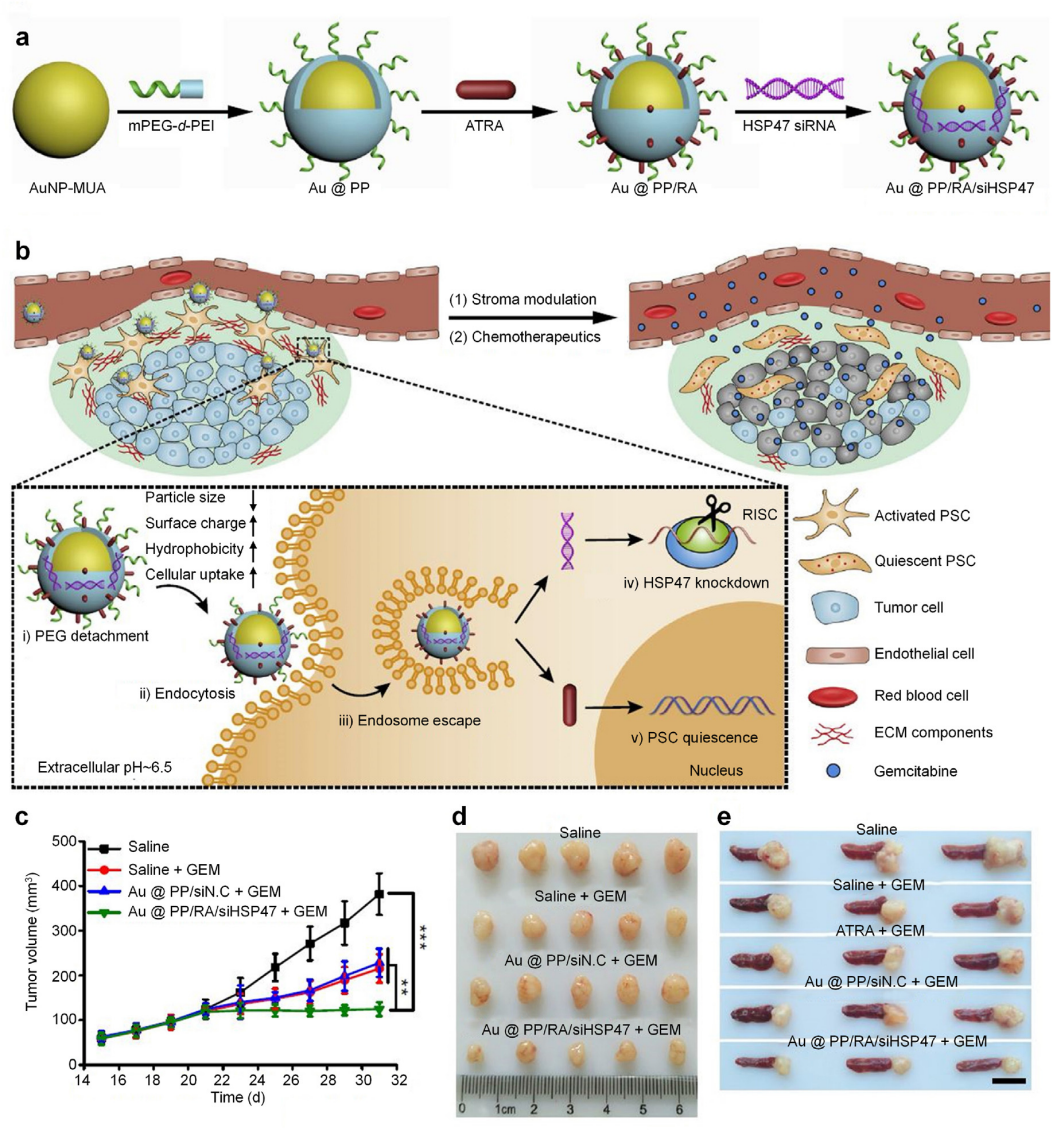
(a) Schematic diagram of the fabrication of the ATRA and HSP47 siRNA codelivery system based on pH-responsive gold nanoparticles. Anionic ATRA and siRNA were electrostatic absorbed onto “sheddable” PEG-grafted PEI-coated AuNP. (b) Schematic diagram of PSCs re-education and tumor ECM modulation by nanosystem for enhanced chemotherapy. The nanosystem is “activated” (PEG shedding, size decrease, charge increase, and hydrophobic ligand exposure) in the acidic pancreatic tumor microenvironment (pHe∼6.5) and exhibits pHe and ATRA dual-enhanced cellular uptake and HSP47 knockdown in PSCs. Consequently, activated PSCs revert to quiescent phenotype and the desmoplastic stroma is homoeostatically restored, with improved blood perfusion and drug delivery. (c) Tumor growth curves during treatment. (d) Image of excised tumors. (e) Image of excised tumors with spleens. Reproduced with permission from Ref. [55]. Copyright 2018, Springer Nature.

To overcome the ECM barrier for drug delivery and penetration in PDAC, Nie's group reported a tumor microenvironment-activated nanosystem based on PEGylated polyethylenimine (PEI)-coated gold nanoparticles (Au@PP) to improve chemotherapeutic efficacy of gemcitabine [55]. Successive integrations of Au@PP with all-trans retinoic acid (ATRA, an inducer of PSC quiescence) and siRNA targeting heat shock protein 47 (HSP47, a collagen-specific molecular chaperone) led to the formation of nanosystem (Au@PP/RA/siHSP47) (Fig. 4a). Under acidic tumor extracellular pH, surface PEG was detached to increase the cellular uptake of nanoparticles by PSCs, in which, ATRA and siHSP47 were released to convert activated PSCs into quiescent phenotype, leading to inhibition of tumor ECM formation (Fig. 4b). As such, the major ECM components were remarkably decreased to improve drug delivery and penetration in 3D PDAC stroma-rich tumor spheroid model and desmoplastic PDAC xenograft tumor model. Such a tumor ECM modulating nanosystem greatly improved the antitumor efficacy of gemcitabine to inhibit the growths of aggressive subcutaneous and orthotropic pancreatic tumors (Fig. 4c–e).

In another study, Nie and colleagues developed a size switchable nanosystem for improving the therapeutic efficacy of PDAC [56]. Paclitaxel (TAX)-incorporated PEG-PLGA nanospheres in hydrophobic layer were encapsulated with hydrophobic vactosertib (VAC)-carrying liposomes and the surface-modified with the fibronectin extra domain B targeting peptide to construct the nanosystems. In a mouse model of orthotopic pancreatic cancer, anchoring of fibronectin extra domain B targeting peptide to abundant tumor-associated fibronectin in ECM promoted the retention of these nanosystems in the tumor stroma after intravenous injection. The subsequent collapse of nanosystems not only released TAX-loaded liposomes with a smaller size for further tumor penetration, but also delivered VAC for inhibiting the expression of ECM proteins, such as fibronectin and collagen. The inhibition of ECM hyperplasia thus promoted more access of TAX to cancer cells in addition to its small size. Such size switchable nanosystem combining ECM modulation overcame the PDAC stromal barrier to drug penetration and led to effective suppression of PANC-1 tumor progression.

Losartan (LOS) is a clinically approved angiotensin II receptor antagonist that can inhibit collagen I production and thus reduces tumor ECM formation [57]. The use of LOS to inhibit ECM formation in tumor tissues for enhanced chemotherapy was reported by Gao's group [58]. 4T1 tumor-bearing mice were pretreated with LOS to reduce the collagen networks in tumor ECM. This improved the penetration and distribution of size shrinkable nanoparticles that were fabricated via conjugating small sized DOX loaded gold nanoparticles onto matrix metalloproteinase-2 (MMP-2) degradable gelatin nanoparticles. After accumulation into tumor tissues, the high-expressed MMP-2 triggered the size shrinkage of nanoparticles and release of DOX. As such, a high intratumoral concentration of DOX was achieved by the co-administration LOS with size shrinkable nanoparticles, leading to higher chemotherapy efficacy in inhibiting the growth of 4T1 tumors in comparison to treatment with nanoparticles alone.

In addition to abovementioned drugs, nitric oxide (NO) has been reported to modulate the tumor ECM to overcome obstacles for enhanced penetration of nanoparticles. As reported by Jin's group, a NO-induced stromal depleting strategy was adopted for enhanced chemotherapy of pancreatic cancer [59]. S-Nitroso-N-acetylpenicillamine (SNAP, a NO donor) loaded liposomes after delivery into PSCs in tumor tissues could suppress the production of ECM via inhibiting the expressions of fibronectin, α-smooth muscle actin and collagen. Such a tumor ECM disruption by NO treatment greatly increased the intratumoral penetration of gemcitabine loaded liposomes, thus significantly improving the drug delivery efficiency into tumors. After sequential delivery of SNAP-loaded liposomes and gemcitabine-loaded liposomes, the tumor growths of both subcutaneous and orthotopic pancreatic tumors in mouse models were remarkably suppressed, suggesting the obviously improved therapeutic efficacy for this NO-induced tumor ECM depleting strategy.

## 3 ECM modulation for enhanced phototherapy

Phototherapy including photothermal therapy (PTT) and photodynamic therapy (PDT) provides a precision treatment strategy for tumor suppression [[60], [61], [62]]. PTT involves the light irradiation of photothermal agents to produce heat for killing cancer cells [63]. PDT utilizes generated ROS during light stimulation of photosensitizers to cause cancer cell death [64]. Both PTT and PDT show unique advantages for cancer treatment, while the dense tumor ECM often obstructs the accumulation and penetration of photothermal agents and photosensitizers into tumor sites, thus greatly compromising the therapeutic efficacy [65,66]. Modulation of tumor ECM is a promising strategy for enhancing the therapeutic efficacy of PTT and PDT.

### 3.1 Tumor ECM degrading strategy

**Fig. 5:**
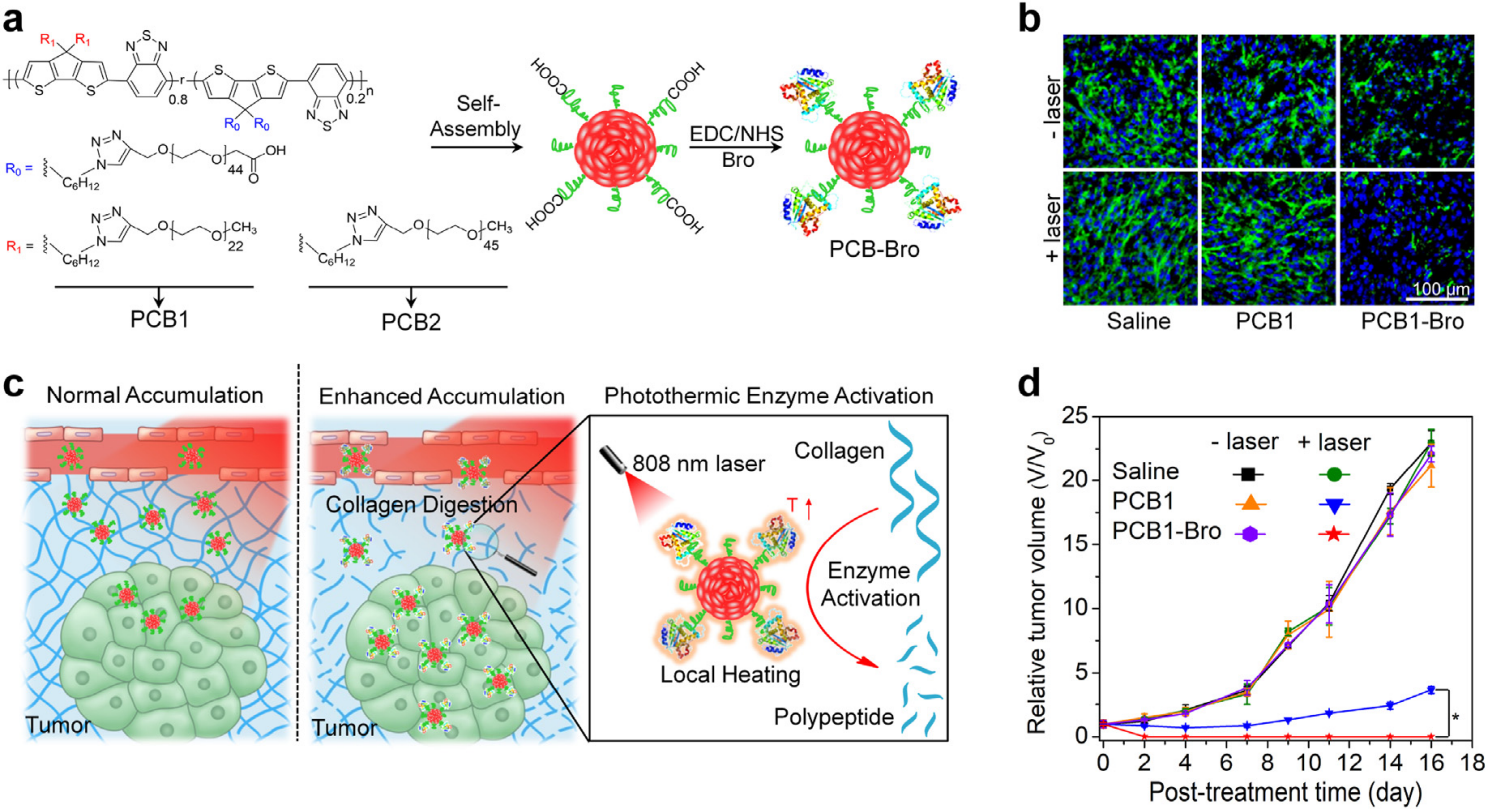
(a) Chemical structures of PCB1 and PCB2 and schematic for the synthesis of PCB-Bro. (b) Immunofluorescence collagen I staining images of 4T1 tumors after intratumoral injection of saline, PCB1, or PCB1-Bro with or without 808 ​nm laser irradiation. (c) Illustration of photothermally triggered enzyme activation of PCB1-Bro towards collagen digestion for enhanced accumulation of nanoparticles in tumor. (d) Tumor growth curves of 4T1 tumor-bearing mice in different groups. Reproduced with permission from Ref. [67]. Copyright 2018, Wiley-VCH.

PTT effect can be enhanced through enzyme-mediated tumor ECM degradation and subsequent improved accumulation of photothermal nanoagents in tumors. For example, Pu's group constructed a polymer nanoenzyme to overcome the nanoparticle penetration obstacle for enhanced cancer PTT [67]. Bromelain with a temperature-responsive enzymatic activity was conjugated onto the surface of poly-(cyclopentadithiophene-alt-benzothiadiazole) (PCB)-based semiconducting polymer nanoparticles to form the polymer nanoenzyme (Fig. 5a). To increase the conjugating efficacy of bromelain, PCB1 with grafting of short-chain methoxy-PEG and long-chain carboxyl-PEG was used. The formed PCB1-Bro effectively accumulated into 4T1 tumor tissues via the enhanced permeability and retention (EPR) effect, in which, PCB1-Bro was irradiated by 808 ​nm laser in a discontinuous manner to produce mild heat (around 42 ​°C) due to PTT effect. The produced heat could obviously improve the enzymatic activity of bromelain to degrade collagen in tumor ECM (Fig. 5b). As such, the accumulation of PCB1-Bro in tumor sites would be further improved (Fig. 5c). Under the second 808 ​nm laser irradiation in a continuous manner, the temperature of tumors for PCB1-Bro treatment was obviously higher than that for PCB1 treatment. Therefore, PCB1-Bro showed a much better PTT efficacy than PCB1 and could completely eradicate 4T1 tumors in living mice (Fig. 5d).

In another study, Ping's group reported a synergistic optical strategy for enhanced deep-tumor of nanoparticles via mediating tumor ECM degradation for enhanced PTT in the second near-infrared (NIR-II) window [68]. Gold nanorods with surface coating of mesoporous polydopamine were utilized to encapsulate papain (a natural protease that can degrade peptides) inside the mesopores. Mesoporous polydopamine coating layer served as a protective shelter for papain to prevent its hydrolysis in tissues. Gold nanorods showed an excellent photothermal efficacy under 1064 ​nm laser irradiation to increase local temperature. Therefore, the temperature-responsive papain could be readily released upon 1064 ​nm laser irradiation, and its enzymatic activity was improved to degrade collagen in tumor ECM. As such, the penetration of photothermal gold nanorods in deep tumors was greatly enhanced. Therefore, the synergistic optical strategy (first mild NIR-II laser irradiation for 15 ​min and then therapeutic NIR-II laser irradiation for 5 ​min) improved the PTT effect of gold nanorods to inhibit HT-29 tumor growth.

The degradation of tumor ECM has also been utilized to improve the efficacy of PDT. As an example, Liu and coworkers used HAase to degrade tumor ECM for enhancing nanoparticle-based PDT of tumors [69]. Administration of free HAase mediated the degradation of HA in tumor ECM, and the tumor vessel densities and effective vascular areas were increased, leading to enhanced tumor uptake of chlorine e6 (Ce6)-conjugated nanomicelles by ∼2-fold. In addition, the tumor oxygenation level was also obviously increased to effectively relieve the hypoxic condition inside tumors. As such, under 660 ​nm laser irradiation, the efficacy of Ce6-conjugated nanomicelle-mediated PDT was significantly improved to inhibit the growth of 4T1 tumors.

### 3.2 Tumor ECM formation inhibiting strategy

Pang's group reported the use of cyclopamine to modulate tumor ECM and thus improve the PTT efficacy of PDAC [70]. Erythrocyte membrane-camouflaged biomimetic gold nanorods with stronger photothermal conversion efficacy and longer *in vivo* circulation than un-camouflaged gold nanorods were developed as the photothermal nanoagents. The treatment of cyclopamine could disrupt the dense tumor ECM and thus significantly increased the accumulation of biomimetic gold nanorods in tumors by 1.8-fold. This led to higher photothermal efficiency in tumor sites under 808 ​nm laser irradiation than the other treatment group. Thus, the combination of cyclopamine treatment with biomimetic gold nanorod-mediated PTT effect achieved the highest efficacy in inhibiting the growth of xenografted Capan-2 tumors.

The therapeutic efficacy of combinational chemotherapy and PTT can also be improved through modulating tumor ECM. As reported by Shen's group, a programmed drug-releasing nanoparticle was developed for tumor ECM remodeling and chemo-photothermal combination therapy of breast cancer [71]. Telmisartan-loaded gelatin nanoparticles were conjugated with platinum nanoparticles attached with PTX through a dual redox responsive diselenide bond to form the drug-releasing nanoparticles. In tumor microenvironment, matrix metalloproteinase-2 mediated the degradation of gelatin to release telmisartan, and diselenide bonds could be destroyed by ROS or glutathione to achieve PTX release. Tumor ECM was degraded due to the released telmisartan-mediated inhibition of transforming growth factor-β (TGF-β) signaling and the direct ablation via photothermal effect of platinum nanoparticles. This allowed enhanced penetration of drug-releasing nanoparticles into deep tumor regions, in which, the released PTX mediated chemotherapy and platinum nanoparticles enabled PTT under 808 ​nm laser irradiation, achieving chemo-photothermal combination therapy. *In vivo* results indicated that these drug-releasing nanoparticles had a significant antitumor effect to inhibit the growth of 4T1 tumors.

In another study, Yuan's group reported a NIR light induced LOS and DOX co-delivery system based on hollow mesoporous prussian blue nanoparticles to degrade tumor ECM and enhance tumor penetration of nanoparticles for synergistic photothermal-chemotherapy [72]. LOS and DOX were loaded in mesoporous prussian blue nanoparticles coated with thermal-responsive lauric acid. Under 808 ​nm laser irradiation, prussian blue nanoparticles mediated PTT effect to produce heat that activated the release of LOS and DOX. Due to the released LOS, tumor ECM was depleted and thus the penetration of nanoparticles and DOX was improved. Via the synergistic action of PTT and chemotherapy, the growth of 4T1 tumors was inhibited by 81.3%.

**Fig. 6:**
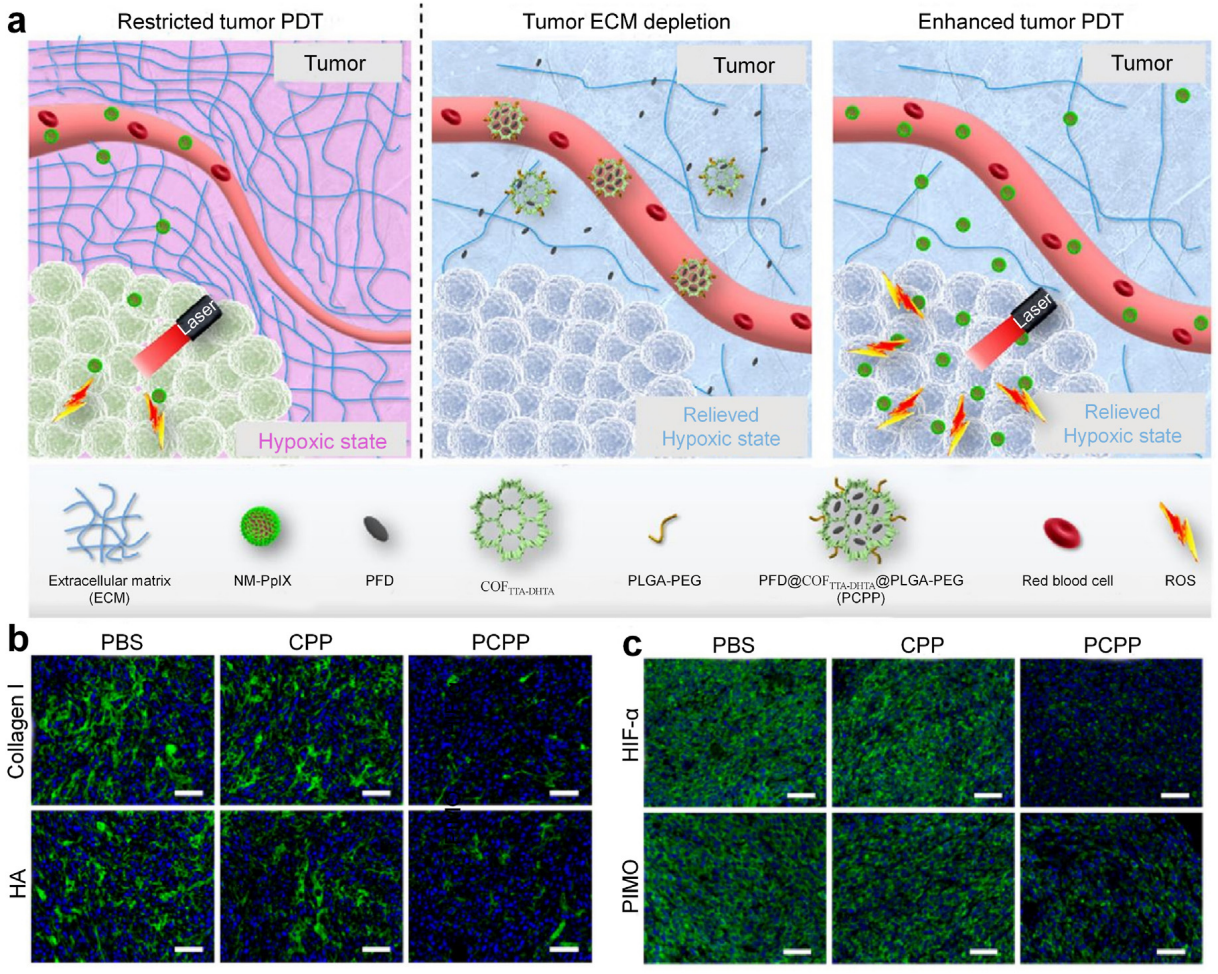
(a) Schematic illustration of PCPP-mediated tumor ECM degradation to enhance tumor PDT effect. 1) Restricted tumor PDT effect due to the insufficient oxygen supply as well as the limited uptake of NM-PpIX in tumor. 2) Selective delivery and release of PFD in tumor tissues by PCPP, PFD-mediated tumor ECM depletion, promotion of tumor vasculature functionality, and alleviation of the hypoxic state of tumor. 3) Enhanced ROS generation and tumor PDT effect due to the improved oxygen supply and tumor uptake of NM-PpIX. (b) Expression of Collagen I and HA in tumors detected by immunocytochemistry of mice after various treatments. (c) Representative fluorescence images of tumor slices from mice with various treatments stained with hypoxia-inducible factor 1-α (HIF-1α) tracer and pimonidazole. Reproduced with permission from Ref. [73]. Copyright 2020, Elsevier.

By utilizing tumor ECM formation inhibition strategy, Zhang and coworkers reported a functional covalent organic framework (COF) to modulate tumor ECM for enhanced PDT (Fig. 6a) [73]. Antifibrotic drug pirfenidone (PFD) was loaded into an imine-based COF containing 4,4′,4’’-(1,3,5-triazine-2,4,6-triyl)trianiline (TTA) and 2,5-dihydroxyterethaldehyde (DHTA) (COF_TTA-DHTA_) with surface decoration of poly(lactic-co-glycolic-acid)-PEG (PLGA-PEG) to fabricate the nanoparticles (PCPP). After accumulation into tumor sites, PCPP released PFD to down-regulate the levels of tumor ECM components, such as collagen I and HA (Fig. 6b). On the one hand, PCPP treatment could improve the accumulation of protoporphyrin IX (PpIX)-conjugated peptide-based nanomicelles (NM-PpIX) serving as the photosensitizer due to the digestion of tumor ECM. On the other hand, PCPP treatment could decompress the tumor blood vessels, restore vascular functionality, thus improving supply of oxygen into tumors to relieve tumor hypoxia (Fig. 6c). The alleviated tumor hypoxia and improved tumor accumulation of nanoagents after PCPP treatment obviously amplified the generation of ROS in tumor sites under 660 ​nm laser irradiation, achieving enhanced PDT effect to eradicate CT26 tumors and inhibit lung metastasis.

## 4 ECM modulation for enhanced immunotherapy

Immunotherapy is a promising treatment strategy for cancer via utilizing the host immune system to kill cancer cells [[74], [75], [76]]. Compared to traditional therapeutic modalities, immunotherapy has unique advantages because of its ability to treat primary and metastatic tumors and prevent tumor recurrence [[77], [78], [79]]. However, the barrier of tumor ECM often limits the penetration of therapeutic agents and infiltrations of immune cells into tumors, and thus the therapeutic efficacy of immunotherapy will be compromised. The modulations of tumor ECM have provided an effective solution to overcome this predicament.

### 4.1 Tumor ECM degrading strategy

To increase the penetration of therapeutic agents into tumors for enhanced immunotherapy, Liu's group reported the use of a pH-responsive HAase-dextran as an adjuvant nanomedicine for enhanced PDT-immunotherapy of cancer [80]. HAase-dextran nanoparticles were dissociated in acidic tumor microenvironment to release native HAase, which led to degradation of HA in tumor ECM to loosen the ECM structure. This would increase the penetration of oxygen, Ce6-loaded liposomes serving as photosensitizers and anti-programmed death-ligand 1 (anti-PD-L1) antibody as immunotherapeutic agents. The enhanced oxygen penetration greatly relieved tumor hypoxia to promote the PDT effect of Ce6-loaded liposomes under 660 ​nm laser irradiation, and reversed the immunosuppressive tumor microenvironment to boost cancer immunotherapy. With HAase-dextran as the adjuvant nanomedicine, the therapeutic efficacy of PDT-combinational anti-PD-L1 checkpoint blockade therapy was significantly enhanced. Both the primary and metastatic CT26 tumors could be treated by this method.

To enhance the tumor infiltration of immune cells for enhanced immunotherapy, Tian's group combined HAase with nanovaccine for treatment of melanoma [81]. The nanovaccine was constructed via co-loading antigen ovalbumin (OVA) and the adjuvant unmethylated cytosine-phosphate-guanine (CpG) into polycationic PEI by electrostatic binding. The positively surface charge of nanovaccines allowed their enhanced uptake by dendritic cells (DCs) to facilitate OVA delivery into the cytoplasm, leading to enhanced maturation of DCs. HAase treatment degraded HA in tumor ECM and thus increased the permeability of tumors, which would enhance the tumor infiltrations of the nanovaccine-generated tumor-specific T cells. High antitumor efficiency was achieved via combining HAase with nanovaccines, and the growth of melanoma was obviously inhibited.

Shen's group reported a microneedle with loading of HAase-modified semiconducting polymer nanoparticles and immune adjuvant polyinosine-polycytidylic acid (PIC) to achieve HAase-mediated tumor ECM degradation and immune cell penetration for effective photothermal-immunotherapy [82]. PIC is a Toll-like receptors 3 (TLR3) agonist that can activate natural killer cells, macrophage, and T cells activities via promoting the maturation of DCs and secretion of interferon-γ (IFN-γ) [83]. After piercing into tumor sites, microneedles were degraded to release HAase-modified semiconducting polymer nanoparticles and PIC. HAase dissolved HA in tumor ECM to promote the penetration of nanoparticles, PIC and immune cells into deeper tumors, in which, the semiconducting polymer nanoparticles mediated PTT under 808 ​nm laser irradiation, and PIC promoted the activation of CD4^+^ and CD8^+^ T cells, thus triggering antitumor immunity. Such microneedle-mediated PTT and immunotherapy combination effect could greatly inhibit the growth of melanoma.

**Fig. 7:**
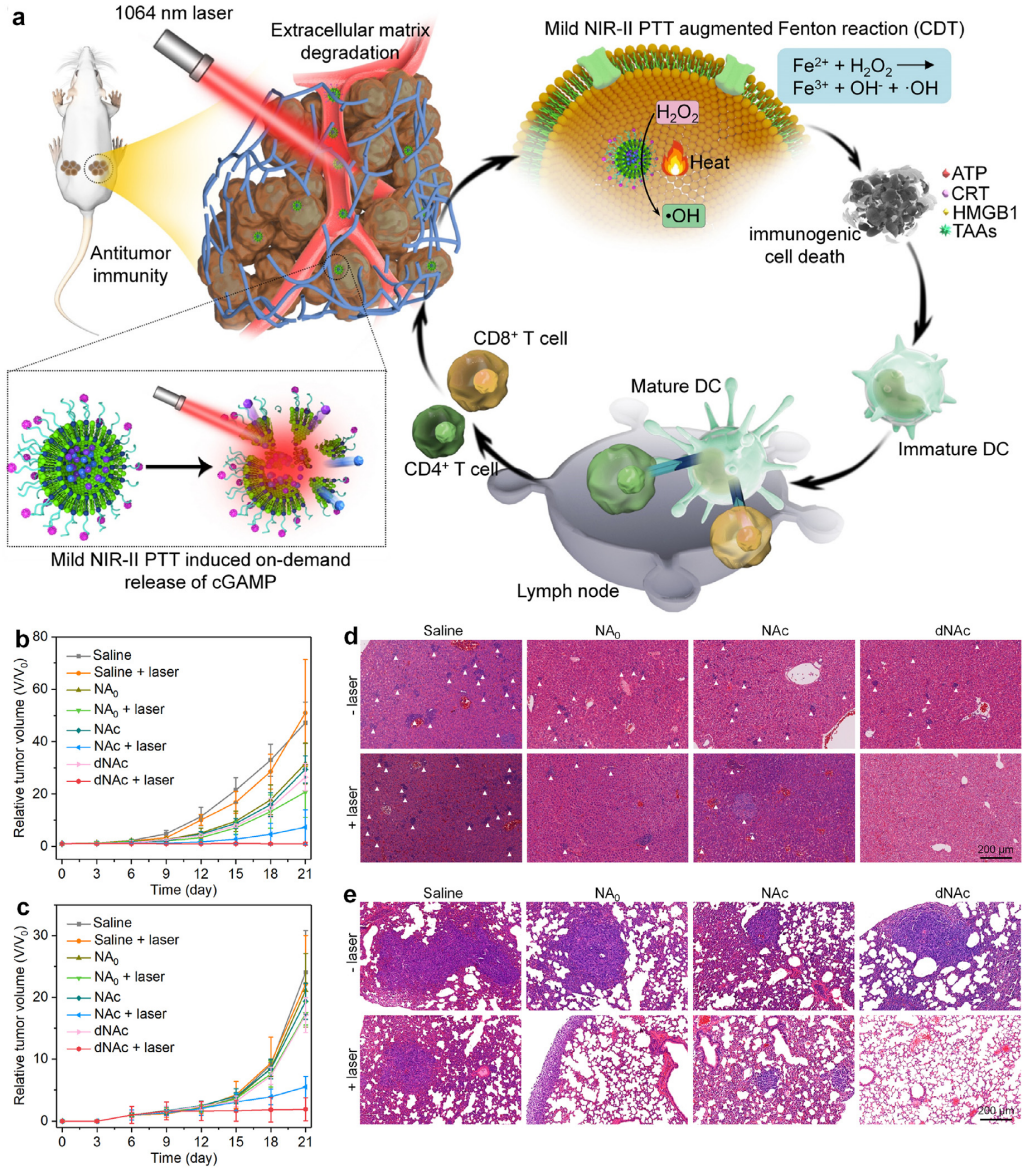
(a) Schematic illustration of NIR-II photoactivation of dNAc for mild photothermal effect-augmented chemodynamic-immunotherapy. (b) Relative tumor volumes of primary tumors from mice after different treatments. (c) Relative tumor volumes of distant tumors from mice after different treatments. (d) Hematoxylin and eosin (H&E) staining images of livers from mice after different treatments. (e) Hematoxylin and eosin (H&E) staining images of lungs from mice after different treatments. Reproduced with permission from Ref. [84]. Copyright 2022, Springer Nature.

In a previous study of Lu's group, an ECM-degrading stimulator of interferon genes (STING) nanoagonist was constructed for mild NIR-II PTT-enhanced chemodynamic-immunotherapy [84]. The thermal-responsive liposomes were utilized to load 2′3′-cyclic guanosine monophosphate-adenosine monophosphate (cGAMP) as STING agonist and ferrous sulfide (FeS_2_) nanoparticles as both NIR-II photothermal agents and Fenton catalysts, and the surface of liposomes was modified with bromelain to construct the nanoagonist (dNAc). Under 1064 ​nm laser irradiation in a discontinuous manner, the nanoagonist generated mild heat (∼45 ​°C) that not only destroyed thermal-responsive liposomes to achieve cGAMP release, but also improved the Fenton reaction efficacy of FeS_2_ nanoparticles to ablate cancer cells and trigger immunogenic cell death (ICD) (Fig. 7a). The in-situ released cGAMP could activate STING pathway to synergize with ICD action, leading to antitumor immunity activation. In addition, the surface modified bromelain could degrade collagen in the tumor ECM to promote the infiltration of effector T cells into tumor tissues. Via such a treatment, the growths of both primary and distant 4T1 tumors were inhibited and the liver and lung metastasis were also obviously suppressed (Fig. 7b–d).

### 4.2 Tumor ECM formation inhibiting strategy

In addition to enzyme-mediated ECM degradation, the inhibition of ECM production has been used for enhanced immunotherapy. David Oupický and coworkers reported a tumor ECM-modulating nanoparticle for the treatment of metastatic pancreatic cancer via improving antitumor immunity [85]. Assembly of anti-miR-210 (to inactivate stroma-producing PSCs) and siKRAS^G12D^ (to kill pancreatic cancer cells) in the cholesterol-modified polymeric CXC motif chemokine receptor type 4 (CXCR4) antagonist nanoparticles led to the formation of the final nanoparticles. The nanoparticles blocked the interaction between cancer cells and stroma to interfere with cancer progression through blocking the binding between CXCR4 and C-X-C motif chemokine ligand 12 (CXCL12) with CXCR4 antagonists. The downregulation of miR-210 and KRAS^G12D^ mediated by anti-miR-210 and siKRAS^G12D^ led to the inactivation of the stroma-producing PSCs and death of pancreatic cancer cells. In addition, the local intraperitoneal administration avoided compromised EPR effect due to low vascular density and dense stroma, and exhibited nearly 15-fold higher tumor aggregation compared to intravenous administration. Due to CXCR4 antagonism and miR-210/KRAS^G12D^ downregulation, such nanoparticles with triple-actions favorably modulated desmoplastic tumor microenvironment and thus promoted the infiltration of cytotoxic T cells into tumor tissues. Such an effective therapy not only directly inhibited the growth and metastasis of orthotopic KPC-derived pancreatic tumors, but also further interfered with cancer progression by disrupting the interaction between cancer cells and stroma.

To facilitate intratumoral T cell infiltration and drug penetration, Wang's group developed a pH responsive nanoparticle to co-deliver TGF-β receptor inhibitor (LY2157299) and siRNA targeting PD-L1 (siPD-L1) for the combination of tumor ECM modulation and antitumor immunotherapy [86]. The nanoparticles formed by self-assembly of PEG-b-poly(ε-caprolactone) (PCL), PCL homopolymer and poly(amidoamine)-graft-PCL (PCL-CDM-PAMAM) were utilized to encapsulate LY2157299 inside, while siPD-L1 was electrostatically adsorbed on the surface by positively charged PAMAM. After accumulation of nanoparticles into the tumor sites, LY2157299 inhibited the activation of PSCs and resulted in a reduction in type I collagen for inhibition of tumor ECM formation. At the same time, the acidic tumor microenvironment triggered the release of small-sized PAMAM that adsorbed siPD-L1, and its penetration into the tumors was significantly facilitated for downregulating the expression of immunosuppressive PD-L1. In addition, the tumor ECM modulation greatly increased the infiltration of CD8^+^ T cells into tumors. Such LY2157299-mediated pancreatic tumor ECM modulation combined with inhibition of PD-L1 checkpoint significantly suppressed the growth of Panc02 tumors.

**Fig. 8:**
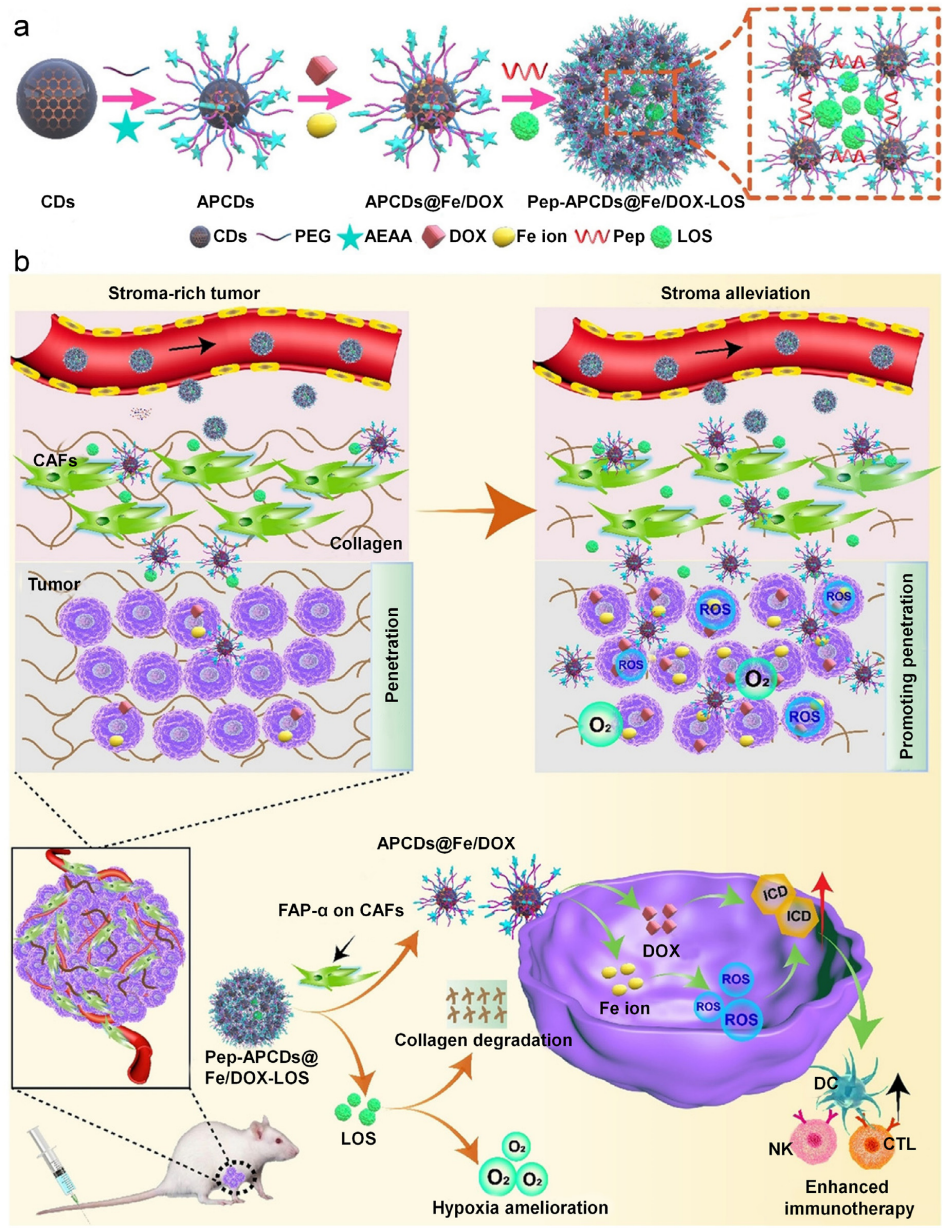
(a) Schematic illustration for the preparation of drugs-loaded nanoassemblies. Small sized CDs are decorated with PEG with and without aminoethyl anisamide (AEAA) (a targeting ligand of sigma receptor) conjugates to obtain APCDs. The APCDs are immobilized with DOX molecules and Fe ions on the surface, and crosslinked by fibroblast activation protein-α (FAP-α)-sensitive Asp-Ala-Thr-Gly-Pro-Ala peptides. Subsequently, LOS (the TME modifier) are encapsulated in the mesoporous nanoassemblies (Pep-APCDs@Fe/DOX-LOS). b) The transformation and enhanced antitumor immunity mechanism of Pep-APCDs@Fe/DOX-LOS. In response to CAFs near blood vessels, the nanoassemblies disassociate into individual APCDs to release LOS to modulate TME by reducing the generation of stromal collagen and improving oxygen perfusion inside tumors. The disassembled APCDs carrying DOX and Fe ions further penetrate into deep tumor sites to induce optimized immunogenic cell death Reproduced with permission from Ref. [87]. Copyright 2021, Wiley-VCH.

The ECM modulation has also been used to enhance the therapeutic efficacy of combinational chemotherapy and immunotherapy. For example, Lu's group reported a CAF-responsive honeycomb-like nanoassembly to regulate multisite delivery for enhanced antitumor chemoimmunotherapy [87]. To obtain such nanoasystems, DOX and immunotherapeutic enhancer (Fe ions) immobilized on the surface of carbon dots (CDs) with modification of aminoethyl anisamide (AEAA, a targeting ligand of sigma receptor) (APCDs) were crosslinked by fibroblast activation protein-α (FAP-α)-responsive Asp-Ala-Thr-Gly-Pro-Ala peptides, followed by encapsulation of tumor microenvironment modifier (LOS) in the mesopores between carbon dots within the nanoassemblies (Fig. 8a). These nanoassemblies could effectively accumulate into the tumors because of the EPR effect and the active targeting to sigma receptors on CAFs mediated by AEAA. Upon response to overexpressed FAP-α on CAFs, the nanoassemblies disassociated into individual CDs to release LOS for reducing intratumoral stromal collagen production, resulting in ECM modulation and tumor hypoxia mitigation (Fig. 8b). The individual CDs carrying DOX and Fe ions exhibited deeper tumor penetration to induce optimized ICD, resulting in intensified immune responses with increased T cell infiltration into tumor sites. Such tumor microenvironment-regulated nanoassemblies combined with synergetic chemoimmunotherapy significantly inhibited 4T1 tumor growth in mouse models.

To further improve the infiltration of immune cells for immunotherapy, Chen's group reported a dual-mechanism based on nano-sapper to simultaneously reduce the physical obstacles of tumor ECM and recruit cytotoxic T cells (CTLs) to potentiate immunotherapy [88]. The nano-sappers consisted an ECM glycoprotein (tenascin C) targeting peptide-decorated calcium phosphate liposome with co-loadings of antifibrotic phosphates-modified α-mangostin and plasmid encoding immune-enhanced cytokine LIGHT (tumor necrosis factor superfamily 14). The surface peptide decoration enhanced the tumor retention of loaded α-mangostin and plasmid. Phosphates-modified α-mangostin reversed the activation of CAFs to decrease collagen production and relieved the compressed vessels. The plasmid allowed the LIGHT expression to stimulate lymphocyte-recruiting chemoattractant expression, which synergized with phosphates-modified α-mangostin to improve the infiltration of CTLs in deep tumors and induced the tertiary lymphoid structures to support the local generation of tumor-specific immune responses. The expressions of α-smooth muscle actin, fibroblast activation protein and fibronectin in tumors were significantly decreased after treatment of nano-sappers. The combination of nano-sappers with anti-programmed death receptor-1 (anti-PD-1) antibody led to an obviously enhanced antitumor efficacy than sole anti-PD-1-mediated immunotherapy in two orthotopic mouse PDAC models.

## 5 Conclusion and perspectives

Dense ECM in tumor tissues is an indispensable component of the tumor microenvironment that plays multiple crucial roles in tumorigenesis, cancer progression, and metastasis [[89], [90], [91]]. In addition, the ECM networks server as the physical barriers to limit the penetration and diffusion of oxygen, therapeutic agents, and effector cells into tumors, leading to significant resistance of different clinical therapeutic modalities. Although tumor blockade therapy, a penetration-independent antitumor modality has been reported [92], the dense ECM is still key barriers for other therapeutic modalities. To overcome these barriers, two major ECM modulating strategies including degrading ECM components and inhibiting the productions of ECM have been adopted. This review summarizes the recent progresses of these modulating strategies to enhance the therapeutic efficacies of nanomedicines. Via modulating the integrality and compactness of tumor ECM, the accumulation, penetration, and diffusion of drug-based nanomedicines, photothermal nanoagents and photosensitizers can be improved, thus greatly enhancing the therapeutic efficacies of chemotherapy, PTT, and PDT. Moreover, tumor ECM modulations can facilitate the infiltration of effector immune cells into solid tumors, which has resulted in amplified immune responses to treat both primary and metastatic tumors.

Despite abovementioned advances, the applications of ECM modulating strategies to enhance the therapeutic outcomes of nanomedicines still have some concerns need to be considered in the future. First, degradation of tumor ECM possibly promotes the migration of tumor cells, which will lead to tumor metastasis even the primary tumors have been well treated. Combination of nanomedicines with anti-metastasis drugs should be a promising strategy to reduce the risks of metastasis [[93], [94], [95]]. Alternatively, NO donors can be used to deliver NO to effectively inhibit tumor metastasis after tumor ECM modulation [96]. Second, the bioavailability of drugs and enzymes that used to modulate tumor ECM need to be increased, and their unwanted accumulations into normal tissues potentially cause side effects. Development of tumor microenvironment- and exogenous stimuli-responsive prodrugs or drug delivery systems can increase the targeting release of these agents in tumor sites [[97], [98], [99], [100], [101]]. Third, nanomedicines are the key components to realize effective cancer treatment, but their clinical uses have not been approved as the *in vivo* long-term safety, biodegradability, and clearance remain questionable. Design of therapeutic nanomedicines with excellent *in vivo* biodegradability or rapid clearance properties will greatly promote their clinical translation [[102], [103], [104], [105]]. Fourth, the physicochemical properties of nanomedicines are important for the interaction with ECM and tortuous transport paths, which should be considered. Smart transformable nanoparticles with self-adjust physicochemical properties can be developed to further improve the diffusion in solid tumors [106]. Fifth, current studies have been only conducted using mouse tumor xenografts, while the ECM properties for these models are much different from those of patient-derived xenograft models [[107], [108], [109]]. Therefore, exploring the feasibilities to combine tumor ECM modulating strategies with nanomedicines for treatments of patient-derived xenograft models are highly recommended [27].

Overall, these tumor ECM modulating strategies have shown great promises to improve the effect of nanomedicines for cancer treatment. Due to the facile design and constructions of nanomedicines, these strategies may be utilized for other therapeutic modalities (such as radiotherapy, sonodynamic therapy, and microwave therapy). Different therapeutic elements (such as photothermal agents, chemotherapeutic drugs, immunotherapeutic agents, photosensitizers, radionuclides and sonosensitizers) can be integrated into a single nanomedicine to enable multimodal therapy, which will help to achieve complete eradications of tumors. In addition, other tumor microenvironment modulating strategy, such as vasculature regulation can be combined with ECM modulation to further enhance the accumulation of nanomedicines [110]. Such a combination of tumor ECM modulating strategies with nanomedicines should be translated into clinical applications for cancer treatment.

## Authors’ contributions

ML and YZ write the original draft manuscript; QZ and JL review and edit the manuscript. All authors read and approved the final manuscript.

## Availability of data and materials

The review is based on the published data and sources of data upon which conclusions have been drawn can be found in the reference list.

## Declaration of competing interest

The authors declare that they have no known competing financial interests or personal relationships that could have appeared to influence the work reported in this paper.

## References

[1] Shaked Y. (2019). The pro-tumorigenic host response to cancer therapies. Nat. Rev. Cancer.

[2] Wyld L., Audisio R.A., Poston G.J. (2015). The evolution of cancer surgery and future perspectives. Nat. Rev. Clin. Oncol..

[3] Qin S.-Y., Cheng Y.-J., Lei Q., Zhang A.-Q., Zhang X.-Z. (2018). Combinational strategy for high-performance cancer chemotherapy. Biomaterials.

[4] Begg A.C., Stewart F.A., Vens C. (2011). Strategies to improve radiotherapy with targeted drugs. Nat. Rev. Cancer.

[5] Chen Q., Wang C., Zhang X., Chen G., Hu Q., Li H., Wang J., Wen D., Zhang Y., Lu Y. (2019). In situ sprayed bioresponsive immunotherapeutic gel for post-surgical cancer treatment. Nat. Nanotechnol..

[6] Markman J.L., Rekechenetskiy A., Holler E., Ljubimova J.Y. (2013). Nanomedicine therapeutic approaches to overcome cancer drug resistance. Adv. Drug Deliv. Rev..

[7] Liu S., Khan A.R., Yang X., Dong B., Ji J., Zhai G. (2021). The reversal of chemotherapy-induced multidrug resistance by nanomedicine for cancer therapy. J. Contr. Release.

[8] De Ruysscher D., Niedermann G., Burnet N.G., Siva S., Lee A.W., Hegi-Johnson F. (2019). Radiotherapy toxicity. Nat. Rev. Dis. Prim..

[9] Borgheti-Cardoso L.N., Viegas J.S.R., Silvestrini A.V.P., Caron A.L., Praca F.G., Kravicz M., Bentley M.V.L.B. (2020). Nanotechnology approaches in the current therapy of skin cancer. Adv. Drug Deliv. Rev..

[10] Hartshorn C.M., Bradbury M.S., Lanza G.M., Nel A.E., Rao J., Wang A.Z., Wiesner U.B., Yang L., Grodzinski P. (2018). Nanotechnology strategies to advance outcomes in clinical cancer care. ACS Nano.

[11] Li T., Shi S., Goel S., Shen X., Xie X., Chen Z., Zhang H., Li S., Qin X., Yang H. (2019). Recent advancements in mesoporous silica nanoparticles towards therapeutic applications for cancer. Acta Biomater..

[12] Wang C., Zhang Z., Chen B., Gu L., Li Y., Yu S. (2018). Design and evaluation of galactosylated chitosan/graphene oxide nanoparticles as a drug delivery system. J. Colloid Interface Sci..

[13] Wang X., Xuan Z., Zhu X., Sun H., Li J., Xie Z. (2020). Near-infrared photoresponsive drug delivery nanosystems for cancer photo-chemotherapy. J. Nanobiotechnol..

[14] Li J., Pu K. (2020). Semiconducting polymer nanomaterials as near-infrared photoactivatable protherapeutics for cancer. Acc. Chem. Res..

[15] Li J., Pu K. (2019). Development of organic semiconducting materials for deep-tissue optical imaging, phototherapy and photoactivation. Chem. Soc. Rev..

[16] Wang X., Zhong X., Liu Z., Cheng L. (2020). Recent progress of chemodynamic therapy-induced combination cancer therapy. Nano Today.

[17] Tian Q., Xue F., Wang Y., Cheng Y., An L., Yang S., Chen X., Huang G. (2021). Recent advances in enhanced chemodynamic therapy strategies. Nano Today.

[18] Hu J.-J., Liu M.-D., Chen Y., Gao F., Peng S.-Y., Xie B.-R., Li C.-X., Zeng X., Zhang X.-Z. (2019). Immobilized liquid metal nanoparticles with improved stability and photothermal performance for combinational therapy of tumor. Biomaterials.

[19] Chen T., Chu Q., Li M., Han G., Li X. (2021). Fe_3_O_4_@Pt nanoparticles to enable combinational electrodynamic/chemodynamic therapy. J. Nanobiotechnol..

[20] Zhang Y., Yang D., Chen H., Lim W.Q., Phua F.S.Z., An G., Yang P., Zhao Y. (2018). Reduction-sensitive fluorescence enhanced polymeric prodrug nanoparticles for combinational photothermal-chemotherapy. Biomaterials.

[21] Xu X., Wu Y., Qian X., Wang Y., Wang J., Li J., Li Y., Zhang Z. (2022). Nanomedicine strategies to circumvent intratumor extracellular matrix barriers for cancer therapy. Adv Healthcare Mater.

[22] Abyaneh H.S., Regenold M., McKee T.D., Allen C., Gauthier M.A. (2020). Towards extracellular matrix normalization for improved treatment of solid tumors. Theranostics.

[23] Zhang Y.R., Lin R., Li H.J., He Wl, Du J.Z., Wang J. (2019). Strategies to improve tumor penetration of nanomedicines through nanoparticle design. Wiley Interdiscip Rev: Nanomed Nanobiotechnol.

[24] Hu J., Yuan X., Wang F., Gao H., Liu X., Zhang W. (2021). The progress and perspective of strategies to improve tumor penetration of nanomedicines. Chin. Chem. Lett..

[25] Zhou Y., Chen X., Cao J., Gao H. (2020). Overcoming the biological barriers in the tumor microenvironment for improving drug delivery and efficacy. J. Mater. Chem. B.

[26] Yang S., Gao H. (2017). Nanoparticles for modulating tumor microenvironment to improve drug delivery and tumor therapy. Pharmacol. Res..

[27] Han X., Xu Y., Geranpayehvaghei M., Anderson G.J., Li Y., Nie G. (2020). Emerging nanomedicines for anti-stromal therapy against desmoplastic tumors. Biomaterials.

[28] Valkenburg K.C., De Groot A.E., Pienta K.J. (2018). Targeting the tumour stroma to improve cancer therapy. Nat. Rev. Clin. Oncol..

[29] Cox T.R. (2021). The matrix in cancer. Nat. Rev. Cancer.

[30] Multhaupt H.A., Leitinger B., Gullberg D., Couchman J.R. (2016). Extracellular matrix component signaling in cancer. Adv. Drug Deliv. Rev..

[31] Overchuk M., Zheng G. (2018). Overcoming obstacles in the tumor microenvironment: recent advancements in nanoparticle delivery for cancer theranostics. Biomaterials.

[32] Zhou Q., Dong C., Fan W., Jiang H., Xiang J., Qiu N., Piao Y., Xie T., Luo Y., Li Z. (2020). Tumor extravasation and infiltration as barriers of nanomedicine for high efficacy: the current status and transcytosis strategy. Biomaterials.

[33] Huo D., Jiang X., Hu Y. (2020). Recent advances in nanostrategies capable of overcoming biological barriers for tumor management. Adv. Mater..

[34] Chen Z., Pan H., Luo Y., Yin T., Zhang B., Liao J., Wang M., Tang X., Huang G., Deng G. (2021). Nanoengineered CAR-T biohybrids for solid tumor immunotherapy with microenvironment photothermal-remodeling strategy. Small.

[35] Wang X., He L., Wei B., Yan G., Wang J., Tang R. (2018). Bromelain-immobilized and lactobionic acid-modified chitosan nanoparticles for enhanced drug penetration in tumor tissues. Int. J. Biol. Macromol..

[36] Wang X., Xu J., Xu X., Fang Q., Tang R. (2020). pH-sensitive bromelain nanoparticles by ortho ester crosslinkage for enhanced doxorubicin penetration in solid tumor. Mater. Sci. Eng. C.

[37] Zinger A., Koren L., Adir O., Poley M., Alyan M., Yaari Z., Noor N., Krinsky N., Simon A., Gibori H. (2019). Collagenase nanoparticles enhance the penetration of drugs into pancreatic tumors. ACS Nano.

[38] Huang H.Y., Chen L.Q., Sun W., Du H.H., Dong S., Ahmed A.M.Q., Cao D., Cui J.H., Zhang Y., Cao Q.R. (2021). Collagenase IV and clusterin-modified polycaprolactone-polyethylene glycol nanoparticles for penetrating dense tumor tissues. Theranostics.

[39] Wang X., Luo J., He L., Cheng X., Yan G., Wang J., Tang R. (2018). Hybrid pH-sensitive nanogels surface-functionalized with collagenase for enhanced tumor penetration. J. Colloid Interface Sci..

[40] Xu F., Huang X., Wang Y., Zhou S. (2020). A size-changeable collagenase-modified nanoscavenger for increasing penetration and retention of nanomedicine in deep tumor tissue. Adv. Mater..

[41] Yao H., Guo X., Zhou H., Ren J., Li Y., Duan S., Gong X., Du B. (2020). Mild acid-responsive "nanoenzyme capsule" remodeling of the tumor microenvironment to increase tumor penetration. ACS Appl. Mater. Interfaces.

[42] Eikenes L., Tari M., Tufto I., Bruland Ø.S., de Lange Davies C. (2005). Hyaluronidase induces a transcapillary pressure gradient and improves the distribution and uptake of liposomal doxorubicin (Caelyx™) in human osteosarcoma xenografts. Br. J. Cancer.

[43] Li G., Fan Y., Lin L., Wu R., Shen M., Shi X. (2021). Two-dimensional LDH nanodisks modified with hyaluronidase enable enhanced tumor penetration and augmented chemotherapy. Sci. China Chem..

[44] Chen E., Han S., Song B., Xu L., Yuan H., Liang M., Sun Y. (2020). Mechanism investigation of hyaluronidase-combined multistage nanoparticles for solid tumor penetration and antitumor effect. Int. J. Nanomed..

[45] Yin S., Gao Y., Zhang Y., Xu J., Zhu J., Zhou F., Gu X., Wang G., Li J. (2020). Reduction/oxidation-responsive hierarchical nanoparticles with self-driven degradability for enhanced tumor penetration and precise chemotherapy. ACS Appl. Mater. Interfaces.

[46] Zhu Q., Chen X., Xu X., Zhang Y., Zhang C., Mo R. (2018). Tumor-specific self-degradable nanogels as potential carriers for systemic delivery of anticancer proteins. Adv. Funct. Mater..

[47] Miao L., Liu Q., Lin C.M., Luo C., Wang Y., Liu L., Yin W., Hu S., Kim W.Y., Huang L. (2017). Targeting tumor-associated fibroblasts for therapeutic delivery in desmoplastic tumors. Cancer Res..

[48] Chauhan V.P., Martin J.D., Liu H., Lacorre D.A., Jain S.R., Kozin S.V., Stylianopoulos T., Mousa A.S., Han X., Adstamongkonkul P. (2013). Angiotensin inhibition enhances drug delivery and potentiates chemotherapy by decompressing tumour blood vessels. Nat. Commun..

[49] Feng J., Xu M., Wang J., Zhou S., Liu Y., Liu S., Huang Y., Chen Y., Chen L., Song Q. (2020). Sequential delivery of nanoformulated α-mangostin and triptolide overcomes permeation obstacles and improves therapeutic effects in pancreatic cancer. Biomaterials.

[50] Chen Y., Song W., Shen L., Qiu N., Hu M., Liu Y., Liu Q., Huang L. (2019). Vasodilator hydralazine promotes nanoparticle penetration in advanced desmoplastic tumors. ACS Nano.

[51] Duong H.T., Dong Z., Su L., Boyer C., George J., Davis T.P., Wang J. (2015). The use of nanoparticles to deliver nitric oxide to hepatic stellate cells for treating liver fibrosis and portal hypertension. Small.

[52] Luo J., Gong T., Ma L. (2020). Chondroitin-modified lipid nanoparticles target the Golgi to degrade extracellular matrix for liver cancer management. Carbohydr. Polym..

[53] Erkan M., Hausmann S., Michalski C.W., Fingerle A.A., Dobritz M., Kleeff J., Friess H. (2012). The role of stroma in pancreatic cancer: diagnostic and therapeutic implications. Nat. Rev. Gastroenterol. Hepatol..

[54] Han H., Hou Y., Chen X., Zhang P., Kang M., Jin Q., Ji J., Gao M. (2020). Metformin-induced stromal depletion to enhance the penetration of gemcitabine-loaded magnetic nanoparticles for pancreatic cancer targeted therapy. J. Am. Chem. Soc..

[55] Han X., Li Y., Xu Y., Zhao X., Zhang Y., Yang X., Wang Y., Zhao R., Anderson G.J., Zhao Y. (2018). Reversal of pancreatic desmoplasia by re-educating stellate cells with a tumour microenvironment-activated nanosystem. Nat. Commun..

[56] Zhao X., Yang X., Wang X., Zhao X., Zhang Y., Liu S., Anderson G.J., Kim S-j, Li Y., Nie G. (2021). Penetration cascade of size switchable nanosystem in desmoplastic stroma for improved pancreatic cancer therapy. ACS Nano.

[57] Diop-Frimpong B., Chauhan V.P., Krane S., Boucher Y., Jain R.K. (2011). Losartan inhibits collagen I synthesis and improves the distribution and efficacy of nanotherapeutics in tumors. Proc. Natl. Acad. Sci. USA.

[58] Cun X., Ruan S., Chen J., Zhang L., Li J., He Q., Gao H. (2016). A dual strategy to improve the penetration and treatment of breast cancer by combining shrinking nanoparticles with collagen depletion by losartan. Acta Biomater..

[59] Chen X., Jia F., Li Y., Deng Y., Huang Y., Liu W., Jin Q., Ji J. (2020). Nitric oxide-induced stromal depletion for improved nanoparticle penetration in pancreatic cancer treatment. Biomaterials.

[60] Jung H.S., Verwilst P., Sharma A., Shin J., Sessler J.L., Kim J.S. (2018). Organic molecule-based photothermal agents: an expanding photothermal therapy universe. Chem. Soc. Rev..

[61] Liu Y., Bhattarai P., Dai Z., Chen X. (2019). Photothermal therapy and photoacoustic imaging via nanotheranostics in fighting cancer. Chem. Soc. Rev..

[62] Lo P.-C., Rodríguez-Morgade M.S., Pandey R.K., Ng D.K., Torres T., Dumoulin F. (2020). The unique features and promises of phthalocyanines as advanced photosensitisers for photodynamic therapy of cancer. Chem. Soc. Rev..

[63] Hu J.-J., Cheng Y.-J., Zhang X.-Z. (2018). Recent advances in nanomaterials for enhanced photothermal therapy of tumors. Nanoscale.

[64] Li X., Lee S., Yoon J. (2018). Supramolecular photosensitizers rejuvenate photodynamic therapy. Chem. Soc. Rev..

[65] Ding M., Zhang Y., Li J., Pu K. (2022). Bioenzyme-based nanomedicines for enhanced cancer therapy. Nano Convergence.

[66] Tan T., Hu H., Wang H., Li J., Wang Z., Wang J., Wang S., Zhang Z., Li Y. (2019). Bioinspired lipoproteins-mediated photothermia remodels tumor stroma to improve cancer cell accessibility of second nanoparticles. Nat. Commun..

[67] Li J., Xie C., Huang J., Jiang Y., Miao Q., Pu K. (2018). Semiconducting polymer nanoenzymes with photothermic activity for enhanced cancer therapy. Angew. Chem. Int. Ed..

[68] Wu D., Chen X., Zhou J., Chen Y., Wan T., Wang Y., Lin A., Ruan Y., Chen Z., Song X. (2020). A synergistic optical strategy for enhanced deep-tumor penetration and therapy in the second near-infrared window. Mater. Horiz..

[69] Gong H., Chao Y., Xiang J., Han X., Song G., Feng L., Liu J., Yang G., Chen Q., Liu Z. (2016). Hyaluronidase to enhance nanoparticle-based photodynamic tumor therapy. Nano Lett..

[70] Jiang T., Zhang B., Shen S., Tuo Y., Luo Z., Hu Y., Pang Z., Jiang X. (2017). Tumor microenvironment modulation by cyclopamine improved photothermal therapy of biomimetic gold nanorods for pancreatic ductal adenocarcinomas. ACS Appl. Mater. Interfaces.

[71] Fang T., Zhang J., Zuo T., Wu G., Xu Y., Yang Y., Yang J., Shen Q. (2020). Chemo-photothermal combination cancer therapy with ROS scavenging, extracellular matrix depletion, and tumor immune activation by telmisartan and diselenide-paclitaxel prodrug loaded nanoparticles. ACS Appl. Mater. Interfaces.

[72] Zhang Y., Liu Y., Gao X., Li X., Niu X., Yuan Z., Wang W. (2019). Near-infrared-light induced nanoparticles with enhanced tumor tissue penetration and intelligent drug release. Acta Biomater..

[73] Wang S.-B., Chen Z.-X., Gao F., Zhang C., Zou M.-Z., Ye J.-J., Zeng X., Zhang X.-Z. (2020). Remodeling extracellular matrix based on functional covalent organic framework to enhance tumor photodynamic therapy. Biomaterials.

[74] Min Y., Roche K.C., Tian S., Eblan M.J., McKinnon K.P., Caster J.M., Chai S., Herring L.E., Zhang L., Zhang T. (2017). Antigen-capturing nanoparticles improve the abscopal effect and cancer immunotherapy. Nat. Nanotechnol..

[75] Li J., Yu X., Jiang Y., He S., Zhang Y., Luo Y., Pu K. (2021). Second near-infrared photothermal semiconducting polymer nanoadjuvant for enhanced cancer immunotherapy. Adv. Mater..

[76] DePeaux K., Delgoffe G.M. (2021). Metabolic barriers to cancer immunotherapy. Nat. Rev. Immunol..

[77] Li J., Luo Y., Pu K. (2021). Electromagnetic nanomedicines for combinational cancer immunotherapy. Angew. Chem. Int. Ed..

[78] Song C., Phuengkham H., Kim Y.S., Lee I., Shin I.W., Shin H.S., Jin S.M., Um S.H., Lee H., Hong K.S. (2019). Syringeable immunotherapeutic nanogel reshapes tumor microenvironment and prevents tumor metastasis and recurrence. Nat. Commun..

[79] Liang J.L., Luo G.F., Chen W.H., Zhang X.Z. (2021). Recent advances in engineered materials for immunotherapy-involved combination cancer therapy. Adv. Mater..

[80] Wang H., Han X., Dong Z., Xu J., Wang J., Liu Z. (2019). Hyaluronidase with pH-responsive dextran modification as an adjuvant nanomedicine for enhanced photodynamic-immunotherapy of cancer. Adv. Funct. Mater..

[81] Guan X., Chen J., Hu Y., Lin L., Sun P., Tian H., Chen X. (2018). Highly enhanced cancer immunotherapy by combining nanovaccine with hyaluronidase. Biomaterials.

[82] He T., Luo Y., Zhang Q., Men Z., Su T., Fan L., Chen H., Shen T. (2021). Hyalase-mediated cascade degradation of a matrix barrier and immune cell penetration by a photothermal microneedle for efficient anticancer therapy. ACS Appl. Mater. Interfaces.

[83] Da Silva C., Camps M., Li T., Chan A., Ossendorp F., Cruz L. (2019). Co-delivery of immunomodulators in biodegradable nanoparticles improves therapeutic efficacy of cancer vaccines. Biomaterials.

[84] Zhan M., Yu X., Zhao W., Peng Y., Peng S., Li J., Lu L. (2022). Extracellular matrix-degrading STING nanoagonists for mild NIR-II photothermal-augmented chemodynamic-immunotherapy. J. Nanobiotechnol..

[85] Xie Y., Hang Y., Wang Y., Sleightholm R., Prajapati D.R., Bader J., Yu A., Tang W., Jaramillo L., Li J. (2020). Stromal modulation and treatment of metastatic pancreatic cancer with local intraperitoneal triple miRNA/siRNA nanotherapy. ACS Nano.

[86] Wang Y., Gao Z., Du X., Chen S., Zhang W., Wang J., Li H., He X., Cao J., Wang J. (2020). Co-inhibition of the TGF-β pathway and the PD-L1 checkpoint by pH-responsive clustered nanoparticles for pancreatic cancer microenvironment regulation and anti-tumor immunotherapy. Biomater. Sci..

[87] Hou L., Chen D., Wang R., Wang R., Zhang H., Zhang Z., Nie Z., Lu S. (2021). Transformable honeycomb-like nanoassemblies of carbon dots for regulated multisite delivery and enhanced antitumor chemoimmunotherapy. Angew. Chem. Int. Ed..

[88] Huang Y., Chen Y., Zhou S., Chen L., Wang J., Pei Y., Xu M., Feng J., Jiang T., Liang K. (2020). Dual-mechanism based CTLs infiltration enhancement initiated by Nano-sapper potentiates immunotherapy against immune-excluded tumors. Nat. Commun..

[89] Kessenbrock K., Plaks V., Werb Z. (2010). Matrix metalloproteinases: regulators of the tumor microenvironment. Cell.

[90] Gu X., Gao Y., Wang P., Wang L., Peng H., He Y., Liu Y., Feng N. (2021). Nano-delivery systems focused on tumor microenvironment regulation and biomimetic strategies for treatment of breast cancer metastasis. J. Contr. Release.

[91] Gerarduzzi C., Hartmann U., Leask A., Drobetsky E. (2020). The matrix revolution: matricellular proteins and restructuring of the cancer microenvironment. Cancer Res..

[92] Jiang Z., Liu Y., Shi R., Feng X., Xu W., Zhuang X., Ding J., Chen X. (2022). Versatile polymer-initiating biomineralization for tumor blockade therapy. Adv. Mater..

[93] Li J., Huang J., Lyu Y., Huang J., Jiang Y., Xie C., Pu K. (2019). Photoactivatable organic semiconducting pro-nanoenzymes. J. Am. Chem. Soc..

[94] Yu W., Hu C., Gao H. (2021). Advances of nanomedicines in breast cancer metastasis treatment targeting different metastatic stages. Adv. Drug Deliv. Rev..

[95] Weber G.F. (2013). Why does cancer therapy lack effective anti-metastasis drugs?. Cancer Lett..

[96] Qin L., Gao H. (2019). The application of nitric oxide delivery in nanoparticle-based tumor targeting drug delivery and treatment. Asian J. Pharm. Sci..

[97] Zhou F., Feng B., Yu H., Wang D., Wang T., Ma Y., Wang S., Li Y. (2019). Tumor microenvironment-activatable prodrug vesicles for nanoenabled cancer chemoimmunotherapy combining immunogenic cell death induction and CD47 blockade. Adv. Mater..

[98] Dong Y., Tu Y., Wang K., Xu C., Yuan Y., Wang J. (2020). A general strategy for macrotheranostic prodrug activation: synergy between the acidic tumor microenvironment and bioorthogonal chemistry. Angew. Chem. Int. Ed..

[99] Ma S., Song W., Xu Y., Si X., Zhang Y., Tang Z., Chen X. (2020). A ROS-responsive aspirin polymeric prodrug for modulation of tumor microenvironment and cancer immunotherapy. CCS Chem.

[100] Phua S.Z.F., Xue C., Lim W.Q., Yang G., Chen H., Zhang Y., Wijaya C.F., Luo Z., Zhao Y. (2019). Light-responsive prodrug-based supramolecular nanosystems for site-specific combination therapy of cancer. Chem. Mater..

[101] Pei Q., Hu X., Zheng X., Liu S., Li Y., Jing X., Xie Z. (2018). Light-activatable red blood cell membrane-camouflaged dimeric prodrug nanoparticles for synergistic photodynamic/chemotherapy. ACS Nano.

[102] Repenko T., Rix A., Ludwanowski S., Go D., Kiessling F., Lederle W., Kuehne A.J. (2017). Bio-degradable highly fluorescent conjugated polymer nanoparticles for bio-medical imaging applications. Nat. Commun..

[103] Lei T., Guan M., Liu J., Lin H.-C., Pfattner R., Shaw L., McGuire A.F., Huang T.-C., Shao L., Cheng K.-T. (2017). Biocompatible and totally disintegrable semiconducting polymer for ultrathin and ultralightweight transient electronics. Proc. Natl. Acad. Sci. U. S. A..

[104] Yu M., Zhou J., Du B., Ning X., Authement C., Gandee L., Kapur P., Hsieh J.T., Zheng J. (2016). Noninvasive staging of kidney dysfunction enabled by renal-clearable luminescent gold nanoparticles. Angew. Chem. Int. Ed..

[105] Chuan L., Zhang J., Yu-Jiao Z., Shu-Fang N., Jun C., Qian W., Shao-Ping N., Ze-Yuan D., Ming-Yong X., Shu W. (2015). Biocompatible and biodegradable nanoparticles for enhancement of anti-cancer activities of phytochemicals. Chin. J. Nat. Med..

[106] Chen J., Jiang Z., Zhang Y.S., Ding J., Chen X. (2021). Smart transformable nanoparticles for enhanced tumor theranostics. Appl. Phys. Rev..

[107] Farace P., Merigo F., Fiorini S., Nicolato E., Tambalo S., Daducci A., Degrassi A., Sbarbati A., Rubello D., Marzola P. (2011). DCE-MRI using small-molecular and albumin-binding contrast agents in experimental carcinomas with different stromal content. Eur. J. Radiol..

[108] Hwang C.I., Boj S.F., Clevers H., Tuveson D.A. (2016). Preclinical models of pancreatic ductal adenocarcinoma. J. Pathol..

[109] Gengenbacher N., Singhal M., Augustin H.G. (2017). Preclinical mouse solid tumour models: status quo, challenges and perspectives. Nat. Rev. Cancer.

[110] Huang X., Ding L., Liu X., Tong R., Ding J., Qian Z., Cai L., Zhang P., Li D. (2021). Regulation of tumor microenvironment for pancreatic cancer therapy. Biomaterials.

